# Synthesis, In Vitro Evaluation and Molecular Docking Studies of Novel Thiophenyl Thiazolyl-Pyridine Hybrids as Potential Anticancer Agents

**DOI:** 10.3390/molecules28114270

**Published:** 2023-05-23

**Authors:** Fayza O. Ashmawy, Sobhi M. Gomha, Magda A. Abdallah, Magdi E. A. Zaki, Sami A. Al-Hussain, Mohamed A. El-desouky

**Affiliations:** 1 Department of Chemistry, Biochemistry Division, Faculty of Science, Cairo University, Giza 12613, Egypt; fayza_othman1@cu.edu.eg; 2 Department of Chemistry, Faculty of Science, Islamic University of Madinah, Madinah 42351, Saudi Arabia; 3 Department of Chemistry, Faculty of Science, Cairo University, Giza 12613, Egypt; drmagdaa725@gmail.com; 4 Department of Chemistry, Faculty of Science, Imam Mohammed Ibn Saud Islamic University (IMSIU), Riyadh 11623, Saudi Arabia; mezaki@imamu.edu.sa (M.E.A.Z.); sahussain@imamu.edu.sa (S.A.A.-H.)

**Keywords:** pyridines, thiazoles, lung cancer, MTT assay, molecular docking, EGFR

## Abstract

Many literature reports revealed the anticancer activity of pyridine and thiazole derivatives, especially in lung cancer. Therefore, a new series of thiazolyl pyridines linked with thiophene moiety via hydrazone group was prepared by one-pot multi-component reaction of (*E*)-1-(4-methyl-2-(2-(1-(thiophen-2-yl)ethylidene)hydrazinyl)thiazol-5-yl)ethanone with benzaldehyde derivatives and malononitrile in a good yield. Then, compound **5** and the thiazolyl pyridines were investigated for their in vitro anticancer activity against lung cancer (A549) cell line using MTT assay compared to doxorubicin as a reference drug. The structure of all the newly synthesized compounds was established based on spectroscopic data and elemental analyses. For better insight to investigate their mechanism of action on A549 cell line, docking studies were performed, targeting epidermal growth factor receptor (EGFR) tyrosine kinase. The results obtained revealed that the tested compounds displayed excellent anticancer activities against lung cancer cell line except **8c** and **8f** compared to reference drug. Based on the data obtained, it can be inferred that the novel compounds, as well as their key intermediate, compound **5**, demonstrated potent anticancer activity against lung carcinoma by inhibiting EGFR.

## 1. Introduction

The world’s most serious affliction, cancer, is the second leading reason for death worldwide. It is one of the life-threatening diseases that impair human health [1]. Globally, in 2020, 19.3 million new cancer cases and 9.9 million cancer-related deaths were recorded, according to the International Agency for Research on Cancer (IARC). By the end of 2030, these numbers are predicted to increase, reaching more than 24.1 million and 13.0 million [2]. Lung cancer, one of the most common types of cancer, is responsible for about 19% of cancer deaths globally, as compared to other types such as stomach, colorectal, hepatic and mammary gland cancers [3]. According to Hanahan and Weinberg, cancer is a diverse disease that displays characteristics associated with uncontrolled growth, proliferation and invasion into other organs and tissues. Cancer cells undergo significant biological changes that give rise to new cellular properties, which are established markers of cancer, including sustained proliferative signaling, growth suppressor inactivation, resistance to apoptosis, increased replicative capacity, promotion of angiogenesis, invasion of neighboring tissues and metastasis [4].

Although chemotherapy is commonly used to treat cancer, it can have significant adverse effects on healthy tissues, resulting in long-term damage to organs such as the heart, lungs, kidneys and reproductive system [5,6].

Numerous investigations have explored the use of diverse sulfur and nitrogen heterocycles, such as thiophene, thiazole, and pyridine, for the treatment of various diseases. Literature suggests that compounds featuring a thiophene core have generated significant attention in the field of drug discovery owing to their potential as anticancer agents, and they operate through multiple pathways implicated in cancer [7,8,9,10,11]. Thiazoles are important heterocyclic compounds due to their diversity of pharmacological properties and significant medicinal utility [12], including anti-bacterial, antimalarial, antiviral, antidiabetic and anticancer activities [13]. Additionally, drugs containing thiazole have been involved in several commercially available remedies for cancer, such as dasatinib [14], patellamide A [15], tiazofurin [16], ixabepilone [17], dabrafenib [18] and epothilone [19]. Moreover, anti-cancer drugs comprising a pyridine ring are streptonigrin, streptonigrone, and lavendamycin. As an attempt to develop a new anti-cancer drug, cytotoxicity and topoisomerase-inhibitory investigations were performed on some pyridine derivatives. Moreover, a large number of pyridine compounds have been recorded for their cardiotonic, antituberculosis, antibacterial and anti-hepatitis B virus activities [20,21].

**Figure 1:**
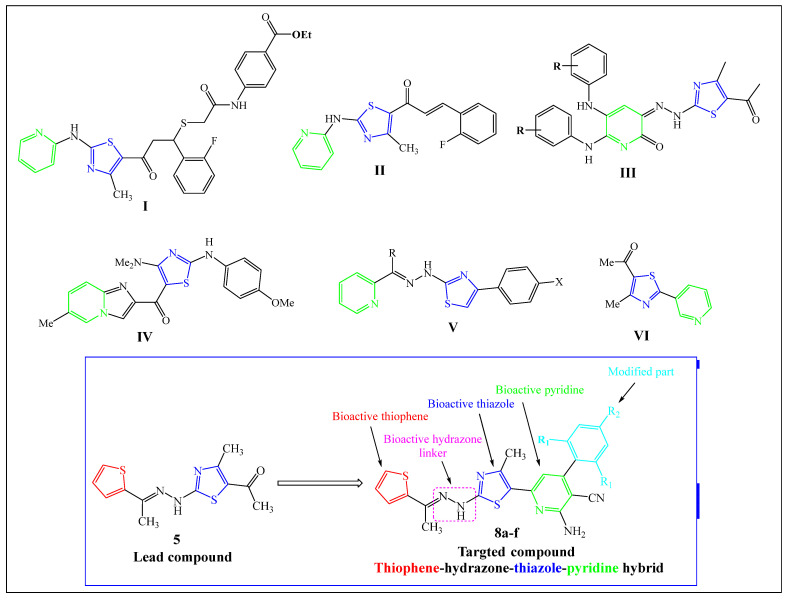
Some thiazole-pyridine hybrids used as potent anticancer agents and the targeted compounds, **8a**–**f**.

Molecular hybridization of two or more heterocyclic rings enhances biological activity, such as antimalarial, tubulin inhibition, antibacterial, antidiabetic and anticancer activities [22,23,24,25,26,27]. Several studies have reported that thiazole-pyridine hybrids exhibit cytotoxicity towards cancer cells and can induce apoptosis in cancer cells (Figure 1) [28,29,30,31,32]. Ivasechko et al. reported the anticancer activity of newly synthesized thiazole-pyridine hybrids (Figure 1, **I** and **II**) against different cancer cells, with compound **I** being the most reactive and sensitive to lung, melanoma, colon and CNS cancer cell lines. Both compounds had shown their ability to affect DNA and cause nuclear morphology alteration that can induce genetic instability in tumor cells. Moreover, the anti-tumor activity of new series of pyridone-thiazole hybrid compounds (Figure 1, **III**) was estimated against colon, gastric and hepatocellular cancer, revealing the most potent one with chlorine atom in ortho position against gastric and hepatic cancer. Moreover, Vasu et al. screened nine imidazo[1,2-a]pyridine-thiazole derivatives for their anticancer activity and showed that hybrid (Figure 1, **IV**) was the most potent inhibitor of NF-ĸB activity. Combining pyridine with five-membered heterocycle containing nitrogen and sulfur (thiazole core) in a single hybrid structure via linker promotes interesting structural and biological characteristics, including anticancer effect on breast, liver and lung cancer (Figure 1, **V**). The pyridine-thiazole hybrid (shown in Figure 1, **VI**) was tested for its antiproliferative activity against various human carcinoma cell lines. It demonstrated notable anti-proliferative activity, specifically against the hepatocyte carcinoma (HEPG2) cell line.

Cancer is associated with the upregulation of epidermal growth factor receptors (EGFRs), potent mediators of normal cell growth and development, along with cell proliferation, apoptosis, angiogenesis and invasion for cancer cells [33]. In normal cells, low EGFR levels were recorded; on the contrary, higher levels were detected in many cancer types as lung, breast, esophagus and colon cancers. Since the amino thiazole derivatives have shown potent EGFR kinase inhibition property, the compounds containing this moiety could be potential EGFR inhibitors for anti-cancer therapy [34].

On the basis of these findings, and in continuation of our previous work on the synthesis of anticancer agents [9,20,35,36,37,38,39,40,41,42] from cheap laboratory-available starting materials with anticipated biological activities, here, hoping to obtain promising molecular candidates as anticancer agents (Figure 1), the aim of this study is to synthesize a novel series of heterocyclic compounds which are hybrids of all the mentioned bioactive heterocyclic moieties.

## 2. Results and Discussion

### 2.1. Chemistry

**Scheme 1:**
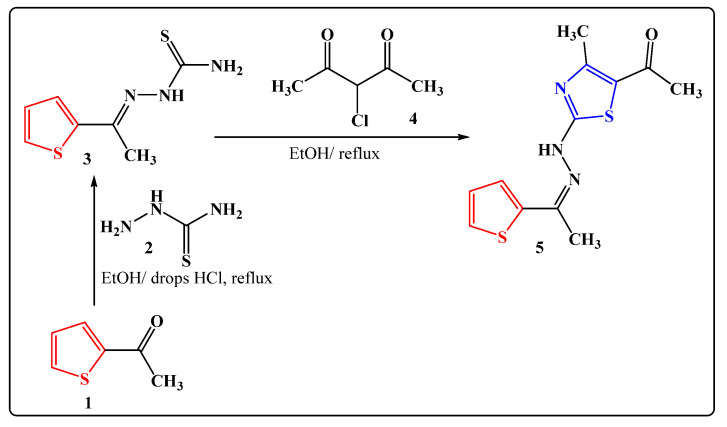
Synthesis of acetylthiazole derivative **5**.

**Scheme 2:**
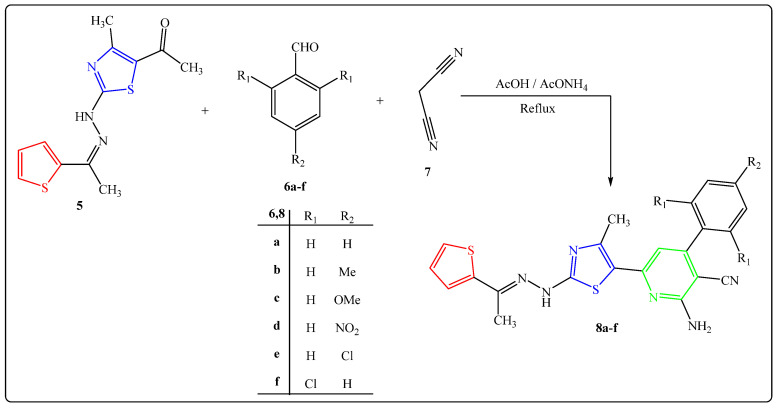
Synthesis of amino cyanopyridine derivatives, **8a**–**f**.

The required starting compound **5**, namely, (*E*)-1-(4-methyl-2-(2-(1-(thiophen-2-yl) ethylidene)hydrazinyl)thiazol-5-yl)ethenone, was prepared as previously reported [43,44,45,46] (Scheme 1) and was used as a building block for preparing a new series of thiazolyl pyridines incorporating thiophene moiety **8a**–**f** via one-pot three-component reaction. Thus, the reaction of compound **5** with benzaldehyde (or its derivatives) **6a**–**f** and malononitrile **7** in the presence of ammonium acetate in glacial acetic acid under reflux led to the formation of products **8a**–**f** (Scheme 2). The structure of the latter compounds, **8a**–**f**, was confirmed by elemental analyses and spectral data. For example, the IR spectra revealed, in each case, two stretching absorption bands in the region ῡ 3148–3204 and 3353–3448 cm^−1^ attributed to the NH, NH_2_ groups, in addition to a sharp band in the region ῡ 2207–2222 cm^−1^ due to the nitrile group. The ^1^H-NMR spectra revealed three singlet signals in the region δ 6.87–6.94, 8.05–8.19 and 8.59–8.81 ppm assigned for the NH_2,_ pyridyl-H and NH protons, respectively, in addition to the multiplet peaks in the aromatic region due to the aromatic protons. The mass spectra for all the new compounds **8a**–**f** revealed, in each case, a molecular ion peak which is in accordance with the respective molecular weight.

**Scheme 3:**
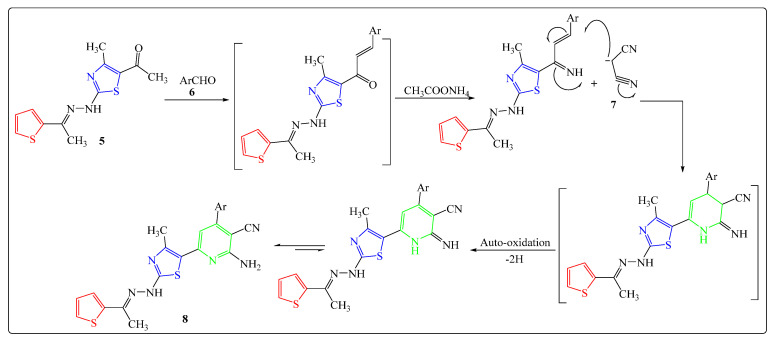
Mechanism of the synthesis of amino cyanopyridine derivatives, **8a**–**f**.

The synthetic pathway of one-pot synthesis of amino cyanopyridine derivatives **8a**–**f** [20,47] starts with the Claisen–Schimdt condensation reaction between the acetylthiazole derivative and substituted benzaldehyde derivatives **6** to form the α, β-unsaturated ketone intermediate. Then, the latter intermediate was reacted with malononitrile through the Michael addition reaction in the presence of ammonium acetate, followed by cyclization, auto-oxidation and finally tautomerization to afford the corresponding pyridine derivatives, **8a**–**f** (Scheme 3).

**Scheme 4:**
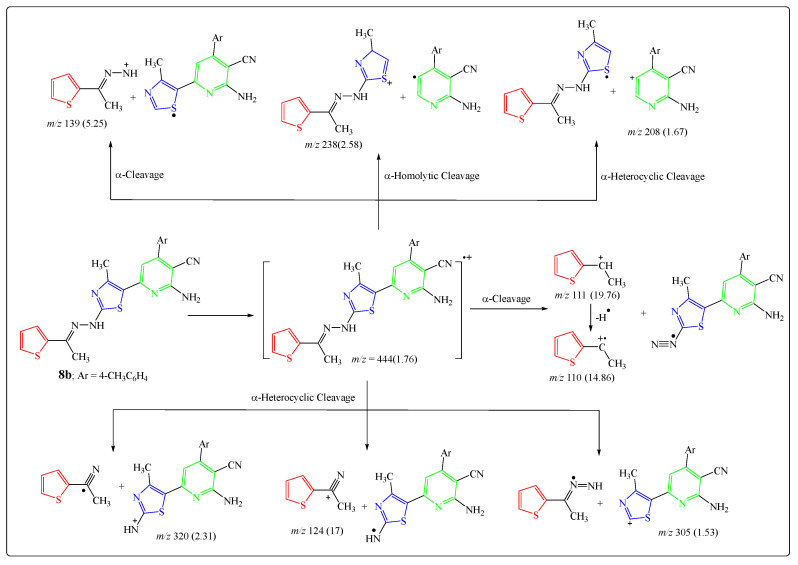
Mass fragmentation pattern of compound **8b**.

The mass spectrum fragmentation of the newly synthesized compound **8b**, taken as a representative example of series **8a**–**f**, was investigated. The mass spectrum of **8b** showed a molecular ion peak at *m/z* = 444 (1.76%), corresponding to its molecular formula C_23_H_20_N_6_S_2_. This molecular ion underwent fragmentation via homolytic and heterolytic α-cleavage, as shown in Scheme 4, to give the following fragment ions (molecular formula, *m/z* (%)): C_17_H_14_N_5_S; *m/z* = 320 (2.31%); C_17_H_13_N_4_S; *m/z* = 305 (1.53%); C_10_H_12_N_3_S_2_; *m/z* = 238 (2.58%); C_13_H_10_N_3_; *m/z* = 208 (1.67%); C_6_H_7_N_2_S; *m/z* = 139 (5.25%); C_6_H_6_NS; *m/z* = 124 (17%); C_6_H_7_S; *m/z* = 111 (19.76%); C_6_H_6_S; *m/z* = 110 (14.86%).

### 2.2. Cytotoxic Activity

The acetylthiazole derivative **5** and the newly synthesized compounds **8a**–**f** were evaluated for their in vitro anticancer screening against human lung cancer (A549) cell line using MTT colorimetric assay with doxorubicin as a reference drug. Data obtained from MTT assay are depicted in Table 1 and show that generally, the tested compounds exert promising cytotoxic activities against A549 cell line except **8c** and **8f** when compared to doxorubicin that has IC_50_ of 0.460 µM. It was noticed that compound **5** has IC_50_ value of 0.452 µM. This potency may be due to the presence of both thiophene and thiazole rings in one hybrid molecule through hydrazone linkage exerting this action on A549 cell line in a good manner, which is reflected as an efficient anticancer agent.

This current result is in agreement with Rodríguez et al. [48] who reported an anticancer activity against the A549 cell line for a compound containing thiophene moiety after 24 h treatment. Similarly, a series of compounds comprising thiazole ring was noted to have anticancer activity after treatment of A549 cell line for 24 h [49].

Regarding compounds **8a**–**f**, compounds **8e** and **8f** were identified as the most and least effective ones against the A549 cell line with IC_50_ of 0.302 and 0.788 µM, respectively, while the rest showed an intermediate activity. The anticancer activity of the newly synthesized products **8a**–**f** can be attributed to the addition of a substituted pyridine ring to structure **5**, elucidating that this addition has an influence on the cytotoxicity against human lung cancer cell line. This finding is in line with Suma et al. [50] who recorded an anticancer activity for a series of compounds containing both pyridine and thiazole rings with IC_50_ ranging from 0.66 to 16.03 µM with the most effective one having IC_50_ of 0.66 µM after 24 h treatment of A549 cell line.

### 2.3. Molecular Docking Studies

To investigate the binding mode of all the tested compounds, **5** and **8a**–**f**, towards EGFR tyrosine kinase domain, molecular docking was conducted, where the results were compared with a known EGFR inhibitor as erlotinib, with binding energy S = −21.9889 Kcal/mol in addition to their corresponding binding energies, as shown in Table 2. It was found that compound **5**, having energy E = 41.2253 Kcal/mol, interacts by the nitrogen atom of hydrazone linkage through hydrogen bond with key amino acid threonine (Thr830) with binding energy S = −20.1495 Kcal/mol and bond length of 2.16 Å, suggesting that threonine is the most reactive amino acid in the protein structure with compound **5** as compared to the series.

**Figure 2:**
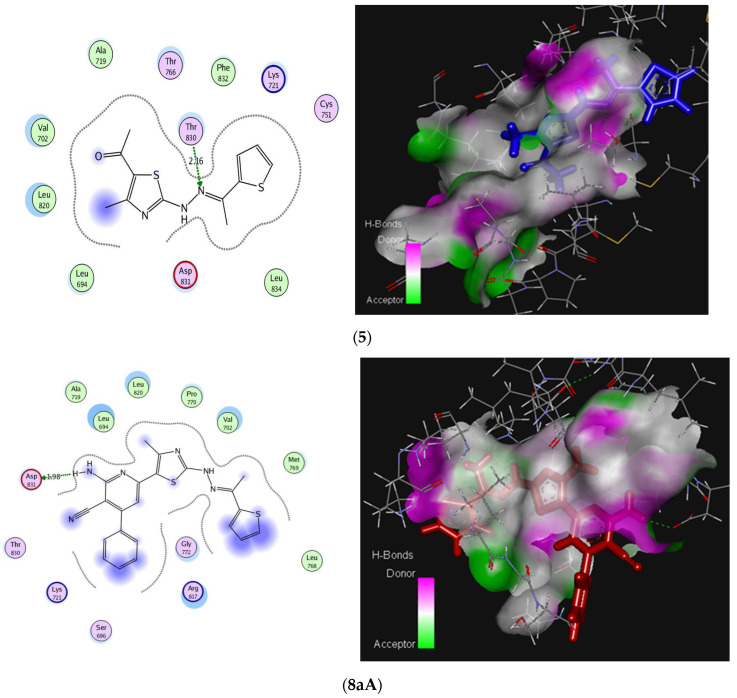

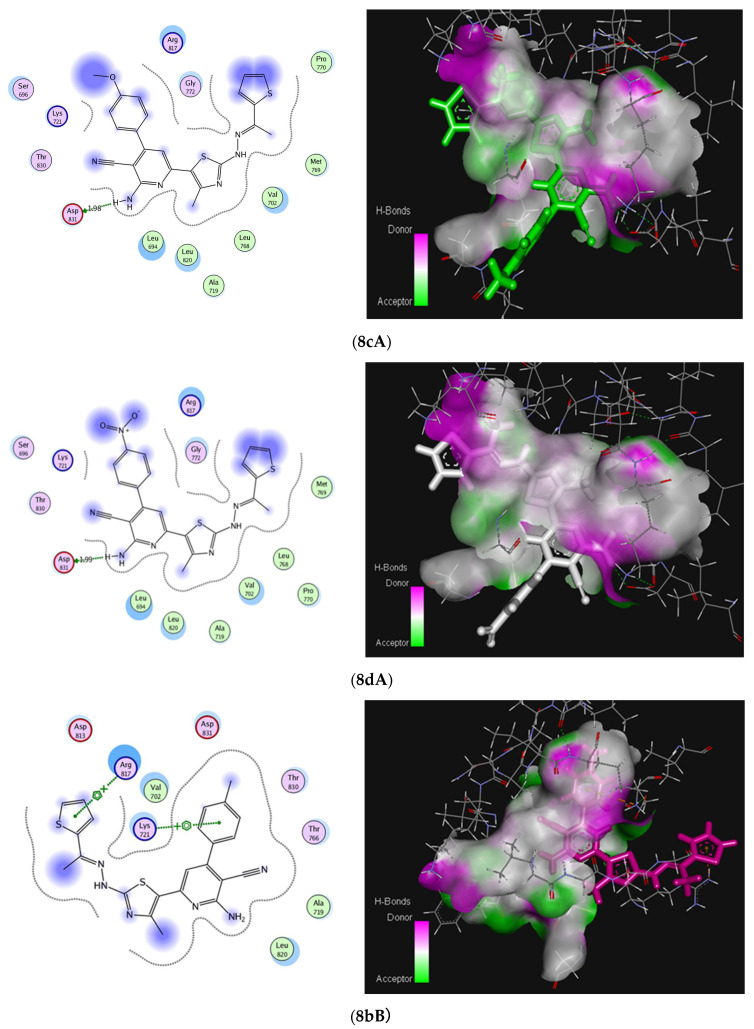

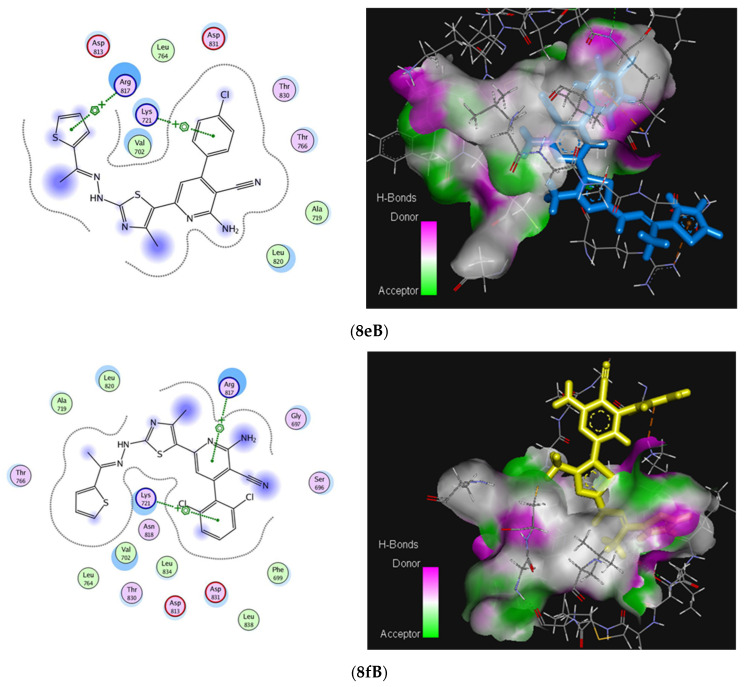
Two-dimensional and three-dimensional depictions of the binding model of EGFR (4HJO) with acetylthiazole derivative **5** and the new compounds, **8a**–**f.** The figures are presented as (**5**): Compound **5**, (**8aA, 8cA, 8dA**)**;** Group A (**8a**,**c**,**d**) and (**8bB, 8eB, 8fB**); and Group B (**8b**,**e**,**f**), respectively.

Based on the interaction of compounds **8a**–**f** with EGFR, as shown in Figure 2, they can be divided into two groups: group A and group B. In group A, which comprises **8a**,**c**,**d** and has mean energy E = 82.7210 Kcal/mol, it was observed that all of them interact via hydrogen bonding (via the hydrogen atom of the amine moiety attached to the pyridine ring) with amino acid Asp831 residue with mean binding energy S = −24.2234 Kcal/mol and mean bond length of 1.989 Å. Meanwhile, group B including **8b**,**e**,**f**, with mean energy E = 75.8467 Kcal/mol and mean binding energy S = −24.4538 Kcal/mol, showed two arene–cation interactions, one between the substituted benzene ring attached to pyridine ring and lysine (Lys721) residue in three compounds and the other between thiophene ring and arginine (Arg817) residue in the case of **8b** and **8e**; except for **8f**, the arene–cation interaction was between pyridine ring and amino acid arginine (Arg817). This change in the interaction may be due to the variance in the molecular weight since **8f** has the highest molecular weight within this group. Concurrently, it was noticed that group B exhibits a slightly more stable interaction with the EGFR active site, which may be due to the difference in molecular weight among all the tested compounds.

## 3. Experimental Section

### 3.1. Reagents Used

2-Acetylthiophene and thiosemicarbazide were purchased from LOBA Chemie (Mumbai, India) and Research-Lab Fine Chem Industries (Mumbai, India), respectively, while both glacial acetic acid and sulfuryl chloride were purchased from Alpha Chemika (Mumbai, India). Ethanol, absolute (HPLC grade) and Acetylacetone were purchased from Fisher Scientific Co. (Loughborough, United Kingdom) and EL Nasr Pharmaceutical Chemical Co. (Cairo, Egypt), respectively. Malononitrile, Penicillin-Streptomycin, 3-(4,5-dimethylthiazol-2-yl)-2,5-diphenyl tetrazolium bromide (MTT) and Trypan blue dye were purchased from Sigma-Aldrich Co. (St. Louis, MO, USA), while Ammonium acetate was purchased from EL Nasr Pharmaceutical Chemical Co. (Egypt). Benzaldehyde, 4-methylbenzaldehyde, 4-chlorobenzaldehyde, 2,6-dichloro benzaldehyde and 4-nitrobenzaldehyde were purchased from Merck (Darmstadt, Germany), while 4-methoxybenzaldehyde and dimethyl sulfoxide (DMSO) and ethanol 70% were purchased from BDH (London, England). Both RPMI 1640 medium with L- glutamine and phosphate buffer saline (PBS) were purchased from Lonza BioWhittaker (Verviers, Belgium), while Fetal bovine serum (FBS) and Trypsin 0.25% were purchased from Gibco (Grand Island, NY, USA) and Amresco (Solon, OH, USA), respectively.

### 3.2. Chemistry

Melting points were measured using an Electrothermal Gallenkamp digital melting point apparatus and were reported uncorrected. IR spectra were recorded in potassium bromide discs using PyeՍnicam SP-1000 spectrometer. ^1^H-NMR spectra were recorded using deuterated dimethyl sulfoxide (DMSO-*d_6_*) solution on a Varian Mercury VX-300 MHz spectrometer, and ^13^C-NMR spectra were recorded at 75.46 MHz. Chemical shifts are quoted in *δ* and were reported related to that of solvents. Mass spectra were recorded using a Shimadzu GCMS-Qp-2010 Plus mass spectrometer (Tokyo, Japan) operating at 70 eV. Elemental analyses were carried out by the Microanalytical Centre of Cairo University, Giza, Egypt.

**Synthesis of 2-(1-(thiophen-2-yl)ethylidene)hydrazine carbothioamide 3** [43,44,45,46]

**Synthesis of (*E*)-1-(4-methyl-2-(2-(1-(thiophen-2-yl)ethylidene)hydrazinyl)thiazol-5-yl) ethanone 5** [43,44,45,46].

**General procedure for synthesis of products 8a–f**

A mixture of compound 5, benzaldehyde or its derivatives **6a**–**f** (2 mmol each) and malononitrile 7 in glacial acetic acid (5 mL) containing 0.5 g of ammonium acetate was refluxed for 4 h and then cooled. The solid formed was filtered and crystallized from acetic acid to give products **8a**–**f** (see Supporting Information File).

**(E)-2-Amino-6-(4-methyl-2-(2-(1-(thiophen-2-yl)ethylidene)hydrazinyl)thiazol-5-yl)-4-phenylnicotinonitrile (8a)**

Yellow color, yield 83%, m.p 117–119 °C, IR (KBr, cm^−1^): 3426, 3181 (NH_2_, NH), 3064 (Ar-H), 2927 (-C-H), 2212 (C≡N), 1625 (C=N), 1565 (C=C_Arom_), 1313 (C–N), 885 (C–S). ^1^H-NMR (300 MHz, DMSO-*d_6_*) *δ* 2.36 (s, 3H, CH_3_), 2.39 (s, 3H, CH_3_), 6.89 (s, 2H, NH_2_), 7.04–7.05 (m, 1H, thienyl-H), 7.37–7.45 (m, 5H, Ar-H), 7.49–7.51 (d, 1H, thienyl-H), 7.64–7.66 (d, 1H, thienyl-H), 8.09 (s, 1H, pyridyl-H), 8.63 (s, 1H, NH) ppm. ^13^C-NMR (DMSO-*d_6_*): δ = 14.7, 21.2 (CH_3_), 112.3, 119.9, 122.1, 126.8, 127.1, 127.6, 128.3, 128.9, 129.3, 129.9, 134.1, 143.5, 144.9, 148.3, 152.3, 156.1, 168.4, 169.2 (Ar-C and C=N) ppm. MS, *m/z* (%): 431 (M^+^, 1), 327 (3), 279 (47), 236 (4.16), 141 (100), 139 (2), 124 (63.7), 111 (14.68), 110 (46.56), 97 (24), 77 (61), 67 (34), 57 (10). Analysis for C_22_H_18_N_6_S_2_ (430.56). Calcd: C, 61.37; H, 4.21; N, 19.52. Found: C, 61.20; H, 3.98; N, 19.30%.

**(*E*)-2-Amino-6-(4-methyl-2-(2-(1-(thiophen-2-yl)ethylidene)hydrazinyl)thiazol-5-yl)-4-(p-tolyl)nicotinonitrile (8b)**

Orange color, yield 80%, m.p 116–118 °C, IR (KBr, cm^−1^): 3419, 3189 (NH_2_, NH), 3034 (Ar-H), 2918 (-C-H), 2221 (C≡N), 1605 (C=N), 1550 (C=C_Arom_), 1369 (C–N), 814 (C–S). ^1^H-NMR (DMSO-*d_6_*) *δ* 2.30 (s, 3H, CH_3_), 2.38 (s, 3H, CH_3_), 2.45 (s, 3H, CH_3_), 6.94 (s, 2H, NH_2_), 7.06–7.08 (m, 1H, thienyl-H), 7.23–7.26 (d, *J* = 8 Hz, 2H, Ar-H), 7.36–7.44 (d, 1H, thienyl-H), 7.52–7.57 (d,1H, thienyl-H), 7.84–7.86 (d, *J* = 8 Hz, 2H, Ar-H), 8.08 (s, 1H, pyridyl-H), 8.64 (s, 1H, NH) ppm. ^13^C-NMR (DMSO-*d_6_*): δ = 14.9, 21.1, 21.6 (CH_3_), 113.5, 114.5, 122.0, 127.0, 127.4, 127.7, 129.5, 129.9, 30.2, 130.8, 140.1, 143.0, 145.7, 146.1, 148.8, 150.5, 161.3, 163.0 (Ar-C and C=N) ppm. MS, *m/z* (%): 444 (M^+^, 1.76), 423 (4), 367 (6), 320 (2.31), 313 (17), 305 (1.53), 239 (15), 238 (2.58), 208 (1.67), 141 (8), 139 (5.25), 124 (17), 111 (19.76), 110 (14.86), 97 (37), 83 (48), 71 (58), 57 (100). Analysis for C_23_H_20_N_6_S_2_ (444.59). Calcd: C, 62.14; H, 4.54; N, 18.90. Found: C, 61.92; H, 4.40; N, 18.60%.

**(*E*)-2-Amino-4-(4-methoxyphenyl)-6-(4-methyl-2-(2-(1-(thiophen-2-yl)ethylidene)hydrazinyl)thiazol-5-yl)nicotinonitrile (8c)**

Orange color, yield 86%, m.p 121–123 °C, IR (KBr, cm^−1^): 3383, 3204 (NH_2_, NH), 3097 (Ar-H), 2928 (-C-H), 2208 (C≡N), 1605 (C=N), 1572 (C=C_Arom_), 1299 (C–N), 828 (C–S). ^1^H-NMR (DMSO-*d_6_*) *δ* 2.31 (s, 3H, CH_3_), 2.46 (s, 3H, CH_3_), 3.83 (s, 3H, OCH_3_), 6.87 (s, 2H, NH_2_), 7.07–7.10 (m, 1H, thienyl-H), 7.12–7.21 (d, *J* = 9 Hz, 2H, Ar-H), 7.58–7.62 (d, 1H, thienyl-H), 7.69–7.73 (d,1H, thienyl-H), 7.84–7.86 (d, *J* = 9 Hz, 2H, Ar-H), 8.05 (s, 1H, pyridyl-H), 8.59 (s, 1H, NH) ppm. MS, *m/z* (%): 460 (M^+^, 6), 441 (6), 374 (4), 349 (5), 336 (10), 306 (24), 265 (8), 236 (4.36), 209 (5), 160 (10), 139 (14), 134 (23), 124 (43.62), 121 (100), 111 (23.39), 110 (35.88), 97 (34), 83 (30), 77 (33), 57 (47). Analysis for C_23_H_20_N_6_S_2_O (460.59). Calcd: C, 59.98; H, 4.38; N, 18.25. Found: C, 59.62; H, 4.51; N, 17.82%.

**(*E*)-2-Amino-6-(4-methyl-2-(2-(1-(thiophen-2-yl)ethylidene)hydrazinyl)thiazol-5-yl)-4-(4-nitrophenyl)nicotinonitrile (8d)**

Orange color, yield 85%, m.p 144–146 °C, IR (KBr, cm^−1^): 3430, 3177 (NH_2_, NH), 3079 (Ar-H), 2931 (-C-H), 2212 (C≡N), 1628 (C=N), 1563 (C=C_Arom_), 1316 (C–N), 846 (C–S). ^1^H-NMR (DMSO-*d_6_*) *δ* 2.38 (s, 3H, CH_3_), 2.44 (s, 3H, CH_3_), 6.91 (s, 2H, NH_2_), 7.04–7.07 (m, 1H, thienyl-H), 7.38–7.40 (d, 1H, thienyl-H), 7.51–7.53 (d, 1H, thienyl-H), 7.88–7.92 (d, *J* = 9 Hz, 2H, Ar-H), 8.19 (s, 1H, pyridyl-H), 8.24–8.27 (d, *J* = 9 Hz, 2H, Ar-H), 8.81 (s, 1H, NH) ppm. ^13^C-NMR (DMSO-*d_6_*): δ = 14.6, 21.0 (CH_3_), 114.1, 119.9, 122.4, 123.7, 124.0, 127.1, 127.4, 127.6, 128.1, 128.3, 142.4, 143.4, 147.4, 148.2, 152.2, 155.1, 168.3, 169.0 (Ar-C and C=N) ppm. MS, *m/z* (%): 475.80 (M^+^, 0.35), 423 (1), 393 (1), 367 (2), 339 (3), 313 (6), 264 (6), 239 (8), 236 (2.99), 171 (4), 141 (14), 139 (6.29), 124 (18.43), 111 (24.32), 110 (21.03), 97 (50), 83 (69), 69 (71), 57 (100). Analysis for C_22_H_17_N_7_S_2_O_2_ (475.55). Calcd: C, 55.57; H, 3.60; N, 20.62. Found: C, 55.68; H, 3.51; N, 19.98%.

**(*E*)-2-Amino-4-(4-chlorophenyl)-6-(4-methyl-2-(2-(1-(thiophen-2-yl)ethylidene)hydrazinyl)thiazol-5-yl)nicotinonitrile (8e)**

Yellow color, yield 84%, m.p 199–201 °C, IR (KBr, cm^−1^): 3448, 3148 (NH_2_, NH), 3028 (Ar-H), 2930 (-C-H), 2222 (C≡N), 1630 (C=N), 1563 (C=C_Arom_), 1312 (C–N), 821 (C–S). ^1^H-NMR (DMSO-*d_6_*) *δ* 2.37 (s, 3H, CH_3_), 2.39 (s, 3H, CH_3_), 6.90 (s, 2H, NH_2_), 7.03–7.07 (m, 1H, thienyl-H), 7.32–7.35 (d, *J* = 9 Hz, 2H, Ar-H), 7.46–7.51 (d, 1H, thienyl-H), 7.66–7.69 (d,1H, thienyl-H), 7.85–7.88 (d, *J* = 9 Hz, 2H, Ar-H), 8.08 (s, 1H, pyridyl-H), 8.64 (s, 1H, NH) ppm. ^13^C-NMR (DMSO-*d_6_*): δ = 14.5, 20.9 (CH_3_), 115.0, 120.4, 121.9, 123.2, 125.8, 127.3, 127.4, 127.8, 128.0, 129.4, 141.5, 143.0, 146.8, 147.9, 150.5, 157.0, 167.4, 168.7 (Ar-C and C=N) ppm. MS, *m/z* (%): 467 (M^+^+2, 1), 465 (M^+^, 3), 373 (8), 340 (7), 292 (16), 236 (5.59), 201 (8), 188 (10), 156 (36), 141 (100), 139 (35.85), 124 (60), 111 (66), 110 (42.35), 75 (72), 55 (94). Analysis for C_22_H_17_N_6_S_2_Cl (465.00). Calcd: C, 56.83; H, 3.69; N, 18.08. Found: C, 57.10; H, 3.52; N, 17.80%.

**(*E*)-2-Amino-4-(2,6-dichlorophenyl)-6-(4-methyl-2-(2-(1-(thiophen-2-yl)ethylidene)hydrazinyl)thiazol-5-yl)nicotinonitrile (8f)**

Brownish red color, yield 82%, m.p 201–203 °C, IR (KBr, cm^−1^): 3353, 3194 (NH_2_, NH), 3096 (Ar-H), 2999, 2928 (-C-H), 2207 (C≡N), 1605 (C=N) 1548 (C=C_Arom_), 1297 (C–N), 827 (C–S). ^1^H-NMR (DMSO-*d_6_*) *δ* 2.31 (s, 3H, CH_3_), 2.48 (s, 3H, CH_3_), 6.89 (s, 2H, NH_2_), 7.08–7.10 (m, 1H, thienyl-H), 7.11–7.22 (m, 3H, Ar-H), 7.59–7.68 (m, 2H, Ar-H), 8.13 (s, 1H, pyridyl-H), 8.68 (s, 1H, NH) ppm. MS, *m/z* (%): 499 (M^+^, 0.07), 467 (0.91), 418 (1), 379 (2), 349 (12), 325 (21), 308 (100), 265 (21), 237 (16), 236 (6.64), 139 (10.48), 134 (30), 124 (12.61), 121 (78), 111 (21.61), 110 (34), 77 (34), 57 (34). Analysis for C_22_H_16_N_6_S_2_Cl_2_ (499.45). Calcd: C, 52.91; H, 3.23; N, 16.83. Found: C, 52.71; H, 3.11; N, 16.62%.

### 3.3. Maintenance of Cell Line

Human lung adenocarcinoma (A549) cell line was obtained from Egyptian holding company for vaccines and sera (VACSERA) (Cell Culture lab., Cairo, Egypt). The cell line was maintained according to Esmail et al. [51].

### 3.4. Cytotoxicity Assay

MTT assay is a sensitive, quantitative and reliable colorimetric method that measures cells^’^ viability. It is based on the fact that the water-soluble substrate 3-(4,5-dimethyl thiazol-2-yl)-2,5-diphenyl tetrazolium bromide (MTT), utilized as 5 mg/mL, can be converted by mitochondrial lactate dehydrogenase enzymes (LDH) into a water-insoluble dark blue formazan in living cells. The insoluble purple formazan product is dissolved into a colored solution by the addition of a solubilization solution as dimethyl sulfoxide (DMSO). This colored solution’s absorbance can be measured using a spectrophotometer at a wavelength between 500 and 600 nm [52].

The cytotoxic effect of the synthesized compounds and the reference medication (doxorubicin) was evaluated using MTT assay. The tested compounds **8a–f** were dissolved in DMSO and used at different concentrations to perform the assay. Precultured cell lines were exposed to the tested materials at 2-fold serial dilutions for 24 h at 37 °C following the decantation of growing media. Microscopically, the treated cell lines were inspected for morphological alterations and detached cells. Phosphate buffer saline (PBS), pH 7.2 ± 0.2, was used to remove dead cells. Residual live cells were treated with 0.5 % MTT stain as 25 µL/well. The plate was incubated for 3–4 h at 37 °C. A total of 0.05 mL of DMSO was used to dissolve formed intra-cytoplasmic MTT formazan crystals for 30 min on a plate shaker. Optical densities were determined using ELISA plate reader (Biotek—8000, USA). Data were reported for three independent experiments and represented as means [53]. The viability percentage was computed as follows: Cell viability percentage = (optical density of treated cells/optical density of untreated cells) × 100 [54]. IC_50_ of the tested compounds were determined using Master –plex-2010 program. Morphological changes were observed 24 h post-treatment using an inverted phase contrast microscope according to Zhang et al. [55].

### 3.5. Molecular Docking Study

Docking studies for the compounds were performed using Molecular Operating Environment (MOE) program, 2009.10 version, to examine ligand–protein interactions at the protein active site. The Protein Data Bank (PDB) was used to download the EGFR protein structure (www.rcsb.org (accessed on 8 March 2023), with PDB code: 4HJO). The grid box patterns were 30.077, 5.987 and 1.141. For preparation of the protein, the standard ligand molecules were removed from the protein’s active site; then, hydrogen atoms were added while maintaining all the heavy atoms in place. Partial charges were calculated using the MMFF94x force field. The program’s final structure was saved as a Pdb file and visualized using BIOVIA Discovery Studio V6.1.0.15350 program, where the target molecule seemed to fit into the protein’s active domain in 3D form.

## 4. Conclusions

Overall result, compound **5** and both groups A and B have nearly the same and higher binding energies than previously reported inhibitors as erlotinib, respectively, indicating that synthesized moieties have a greater binding ability with EGFR protein, and resulting in more effective blockage. Based on these findings, compound **5** and amino cyanopyridine derivatives **8a**–**f** were observed to have promising chemotherapeutic effect on lung cancer cells. Compound **5** has the smallest molecular weight, while compounds **8a**–**f** have higher molecular weight. The observed interactions may be accountable for the EGFR inhibition potency of all tested compounds that can vary according to the molecular weight. Also, the tested compounds record better anticancer activity except **8c** and **8f** as compared to the reference drug. As a result, the current investigation indicated that the described thiazolyl pyridines are promising EGFR inhibitors and pave the way for the synthesis of other libraries based on the reported scaffold, which may eventually result in the creation of an effective therapy for lung cancer.

## Figures and Tables

**Table 1:** Evaluation of IC_50_ values (µM) for compounds **5** and **8a**–**f** against A549 cell line.

Compound	IC_50_ (µM)
**5**	0.452
**8a**	0.428
**8b**	0.412
**8c**	0.549
**8d**	0.433
**8e**	0.302
**8f**	0.788
**D** **oxorubicin**	0.460

**Table 2:** Docking interaction with EGFR of all the tested compounds, **5** and **8a**–**f**.

	Compound(s)	Compound 5	Group A (8a,c,d)	Group B (8b,e,f)
Parameter				
E (Kcal/mol) *^a^*		41.2253	82.7210	75.8467
S (Kcal/mol) *^a^*		−20.1495	−24.2234	−24.4538
Residues involved in interaction		Thr830 (H-bond)	Asp831 (H-bond)	Lys721, Arg817 (Arene-cation)
No. of hydrogen bonding		1	1	-
Bond length [Å]		2.16	1.989	-

*^a^* E: Energy of the compound (Kcal/mol); S: Binding energy of compound with EGFR (Kcal/mol). The results for the groups (A and B) are recorded as mean values.

## Data Availability

The data presented in this study are available on request.

## Supplementary Materials

The following supporting information can be downloaded at: https://www.mdpi.com/article/10.3390/molecules28114270/s1, Some ^1^H-, ^13^C-NMR, IR, and Mass spectra of the synthesized compounds.

## Sample Availability

Samples of compounds **5** and **8a**–**f** are available from the authors.
