# Supplementary material for: Synthesis, In Vitro Evaluation and Molecular Docking Studies of Novel Thiophenyl Thiazolyl-Pyridine Hybrids as Potential Anticancer Agents
Source: Molecules. 2023 May 23;28(11):4270. doi: 10.3390/molecules28114270 (PMC10254479; doi:10.3390/molecules28114270)
Supplement: Supplementary file 1 [file molecules-28-04270-s001.zip › molecules-2267479-supplementary.pdf]

Synthesis, In vitro Evaluation and Molecular Docking Studies of Novel Thiophenyl Thiazolyl-Pyridine Hybrids as Potential Anticancer Agents

Fayza O. Ashmawy [1], Sobhi M. Gomha [2,3*], Magda A. Abdallah [3], Magdi E. A. Zaki [4], Sami A. Al-Hussain [4] and Mohamed A. El-desouky [1,*]

1 Department of Chemistry, Biochemistry Division, Faculty of Science, Cairo University, Giza, Egypt; fayza_othman1@cu.edu.eg, meldesouky@sci.cu.edu.eg

2 Department of Chemistry, Faculty of Science, Islamic University of Madinah, Madinah 42351, Saudi Arabia

3 Department of Chemistry, Faculty of Science, Cairo University, Giza, Egypt; drmagdaa725@gmail.com

4 Department of Chemistry, Faculty of Science, Imam Mohammed Ibn Saud Islamic University (IMSIU), Riyadh 11623, Saudi Arabia; mezaki@imamu.edu.sa (M.E.A.Z.); sahussain@imamu.edu.sa (S.A.A.-H.)

* Correspondence: smgomha@iu.edu.sa (S.M.G.); meldesouky@sci.cu.edu.eg (M.A.E.-D.)

27-Aug-19 14:35:41

Cairo University
Micro Analytical Center

DI Analysis
Shimadzu Qp-2010 Plus

Sample Information		Method	
Analyzed by	Dr. Mai Younis	====== Analytical Line 1 ======	
Analyzed	27/08/2019 02:29:38 م	IonSourceTemp	250.00 °C
Sample Name	F2	[MS Table]	
Sample ID		--Group 1 - Event 1--	
Customer Name	Dr. Fayza Othman - Science - Cairo	Start Time	0.00min
Data File	C:\GCMSsolution\Data\Project1\F2.QGD	End Time	10.00min
Org Data File	C:\GCMSsolution\Data\Project1\F2.QGD	ACQ Mode	Scan
Method File	C:\GCMSsolution\Data\Project1\High Temperature Op	Event Time	0.50sec
Org Method File	C:\GCMSsolution\Data\Project1\High Temperature Op	Scan Speed	1111
Report File		Start m/z	50.00
Tuning File	C:\GCMSsolution\System\Tune1_default.qgt	End m/z	550.00
$EndIf$Modified by	Dr. Mai Younis	Electron Voltage	70 eV
Modified	27/08/2019 02:34:32 م	Ionization Mode	EI

C:\GCMSsolution\Data\Project1\F2.QGD

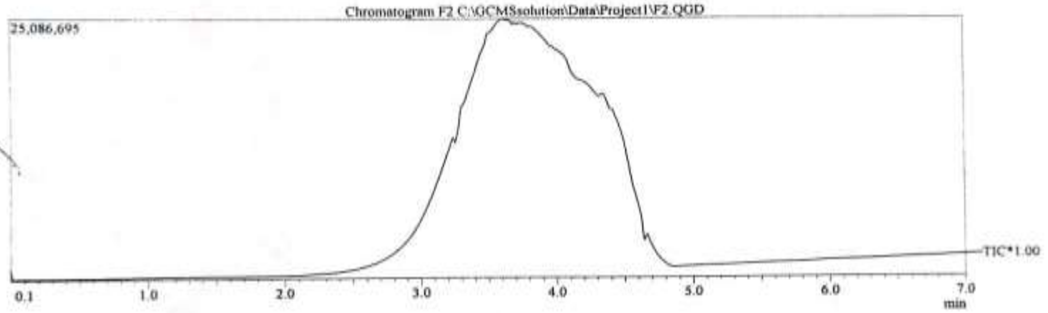

Spectrum

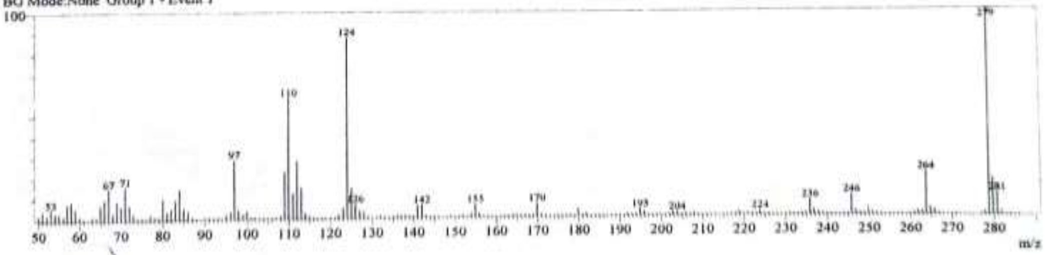

Mass Table
Line#:1 R.Time:3.6(Scan#:436)
MassPeaks:231
RawMode:Single 3.6(436) BasePeak:279(3264752)
BG Mode:None Group 1 - Event 1

#	m/z	Abs. In	Rel. Int.	#	m/z	Abs. In	Rel. Int.	#	m/z	Abs. In	Rel. Int.
1	50.00	60822	1.86	4	53.00	165374	5.07	7	56.05	60482	1.85
2	51.00	140705	4.31	5	54.00	109041	3.34	8	56.95	244535	7.49
3	52.00	82143	2.52	6	55.00	101590	3.11	9	57.95	285652	8.75

Mass spectra of compound **5**

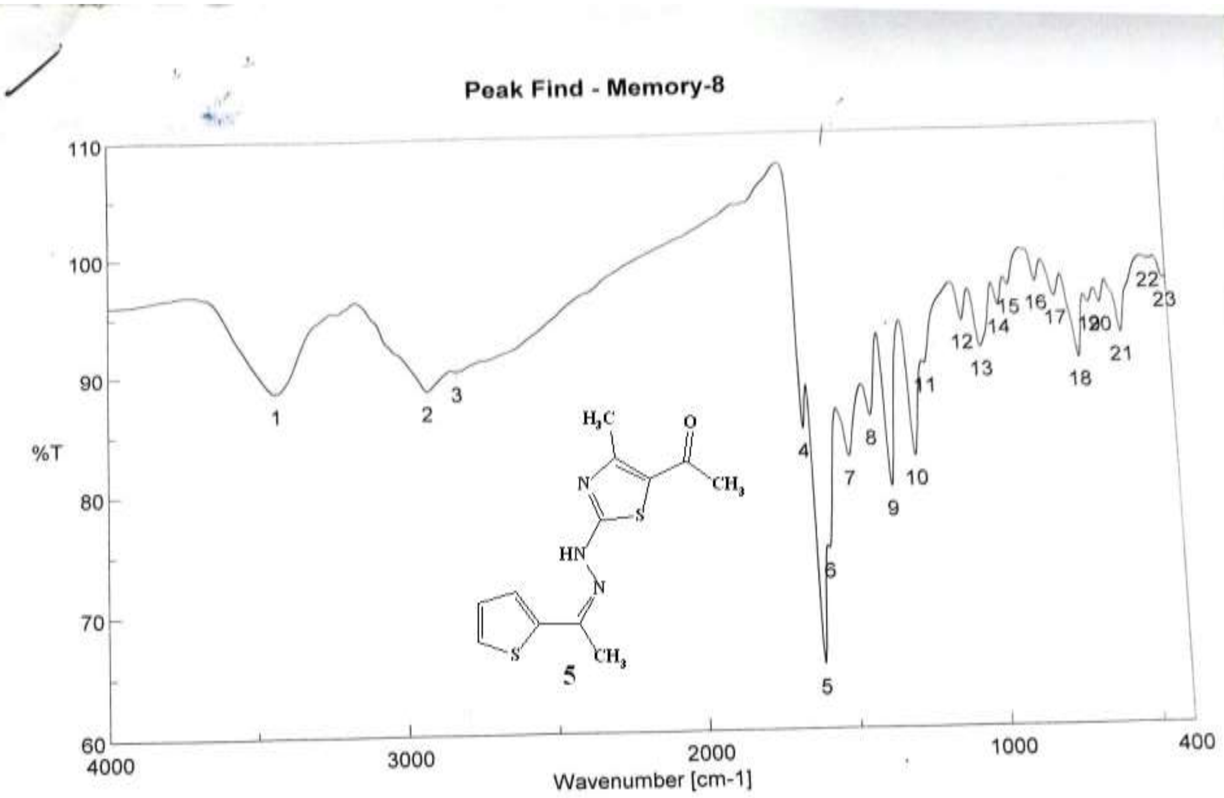

[Comments]
Sample name F2
Comment 12/2018
User IR
Division IR
Company MAC

[Result of Peak Picking]

No.	Position	Intensity	No.	Position	Intensity	No.	Position	Intensity
1	3430.74	88.5809	2	2918.73	88.5304	3	2818.45	90.0999
4	1650.77	84.7602	5	1609.31	65.2476	6	1579.41	74.7496
7	1501.31	82.3557	8	1424.17	85.6966	9	1361.5	79.7901
10	1278.57	82.2721	11	1235.18	89.9593	12	1103.08	93.5253
13	1043.3	91.2129	14	976.769	94.7092	15	940.128	96.3989
16	845.633	96.6811	17	782.958	95.3875	18	711.604	90.1341
19	668.214	94.8291	20	630.609	94.7651	21	564.077	92.1955
22	458.975	98.4484	23	405.942	96.7336			

IR spectra of compound **5**

27-Aug-19 14:43:09

ع. ز. محمود

Cairo University
Micro Analytical Center

DI Analysis
Shimadzu Qp-2010 Plus

Sample Information

Analyzed by	Dr. Mai Younis
Analyzed	27/08/2019 02:37:16 م
Sample Name	F3
Sample ID	
Customer Name	Dr. Fayza Othman - Science - Cairo
Data File	C:\GCMSsolution\Data\Project1\F3.QGD
Org Data File	C:\GCMSsolution\Data\Project1\F3.QGD
Method File	C:\GCMSsolution\Data\Project1\High Temperature Op
Org Method File	C:\GCMSsolution\Data\Project1\High Temperature Op
Report File	
Tuning File	C:\GCMSsolution\System\Tune1_default.qgt
$EndIf$Modified by	Dr. Mai Younis
Modified	27/08/2019 02:41:35 م

Method

===== Analytical Line 1 =====

IonSourceTemp	:250.00 °C
[MS Table]	
--Group 1 - Event 1--	
Start Time	:0.00min
End Time	:10.00min
ACQ Mode	:Scan
Event Time	:0.50sec
Scan Speed	:1111
Start m/z	:50.00
End m/z	:550.00
Electron Voltage	: 70 eV
Ionization Mode	: EI

C:\GCMSsolution\Data\Project1\F3.QGD

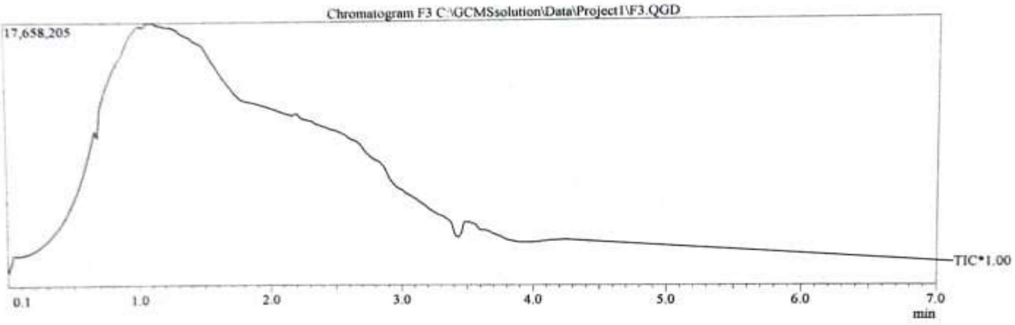

Spectrum

Line#:1 R.Time:2.5(Scan#:298)
MassPeaks:302
RawMode:Single 2.5(298) BasePeak:141(764329)
BG Mode:None Group 1 - Event 1

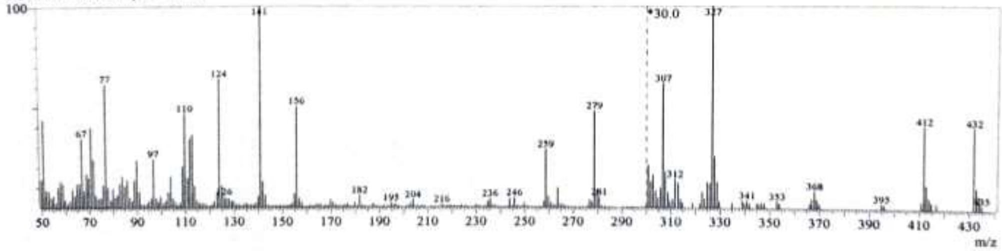

Mass Table
Line#:1 R.Time:2.5(Scan#:298)
MassPeaks:302
RawMode:Single 2.5(298) BasePeak:141(764329)
BG Mode:None Group 1 - Event 1

#	m/z	Abs. In	Rel. Int.	#	m/z	Abs. In	Rel. Int.	#	m/z	Abs. In	Rel. Int.
1	50.00	106316	13.91	4	53.00	63658	8.33	7	56.05	19283	2.52
2	51.00	334652	43.78	5	54.00	35881	4.69	8	57.00	77417	10.13
3	52.00	65186	8.53	6	55.00	42828	5.60	9	57.95	101714	13.31

Mass spectra of compound **8a**

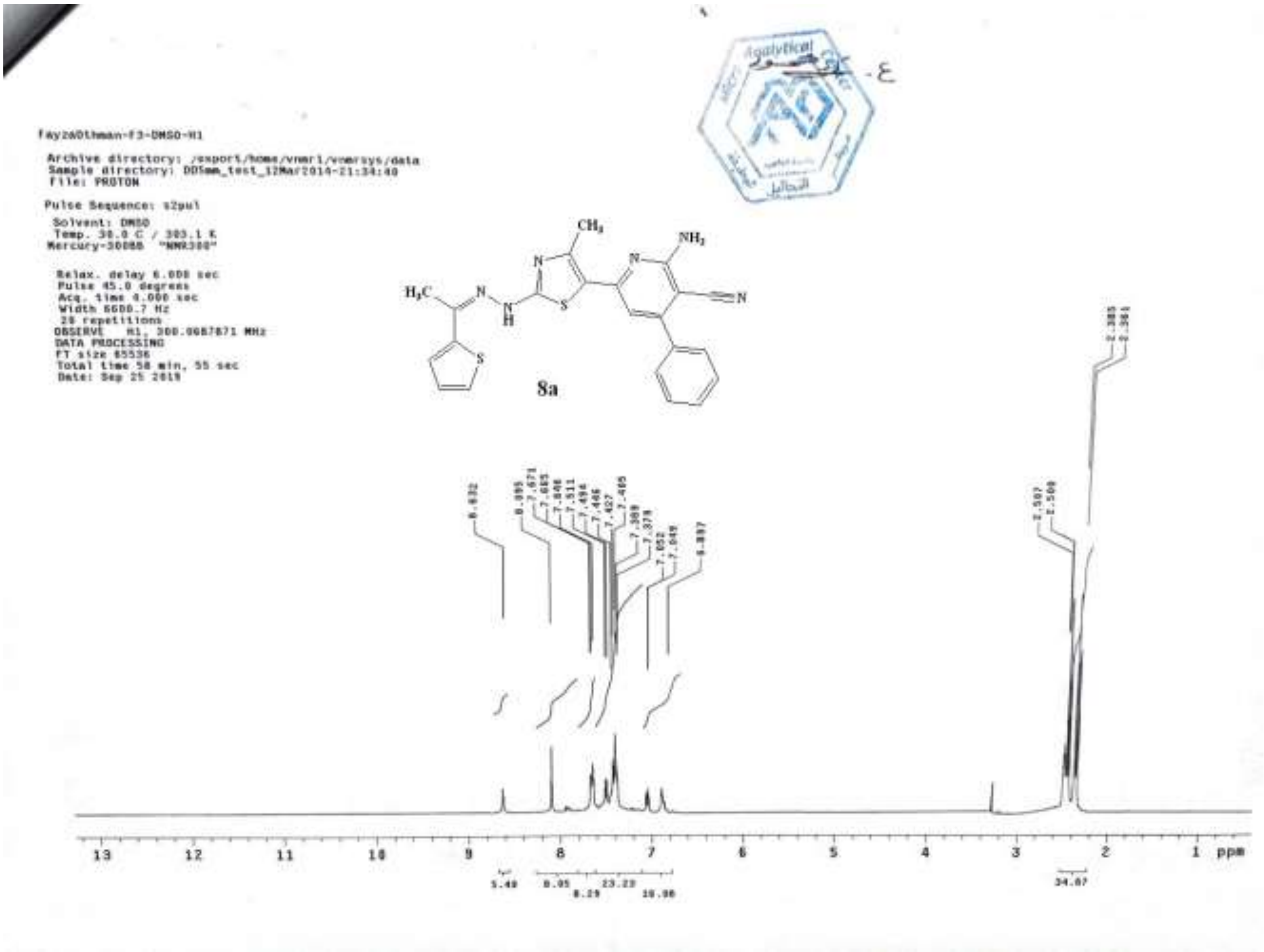

^{1}H-NMR spectra of compound **8a**

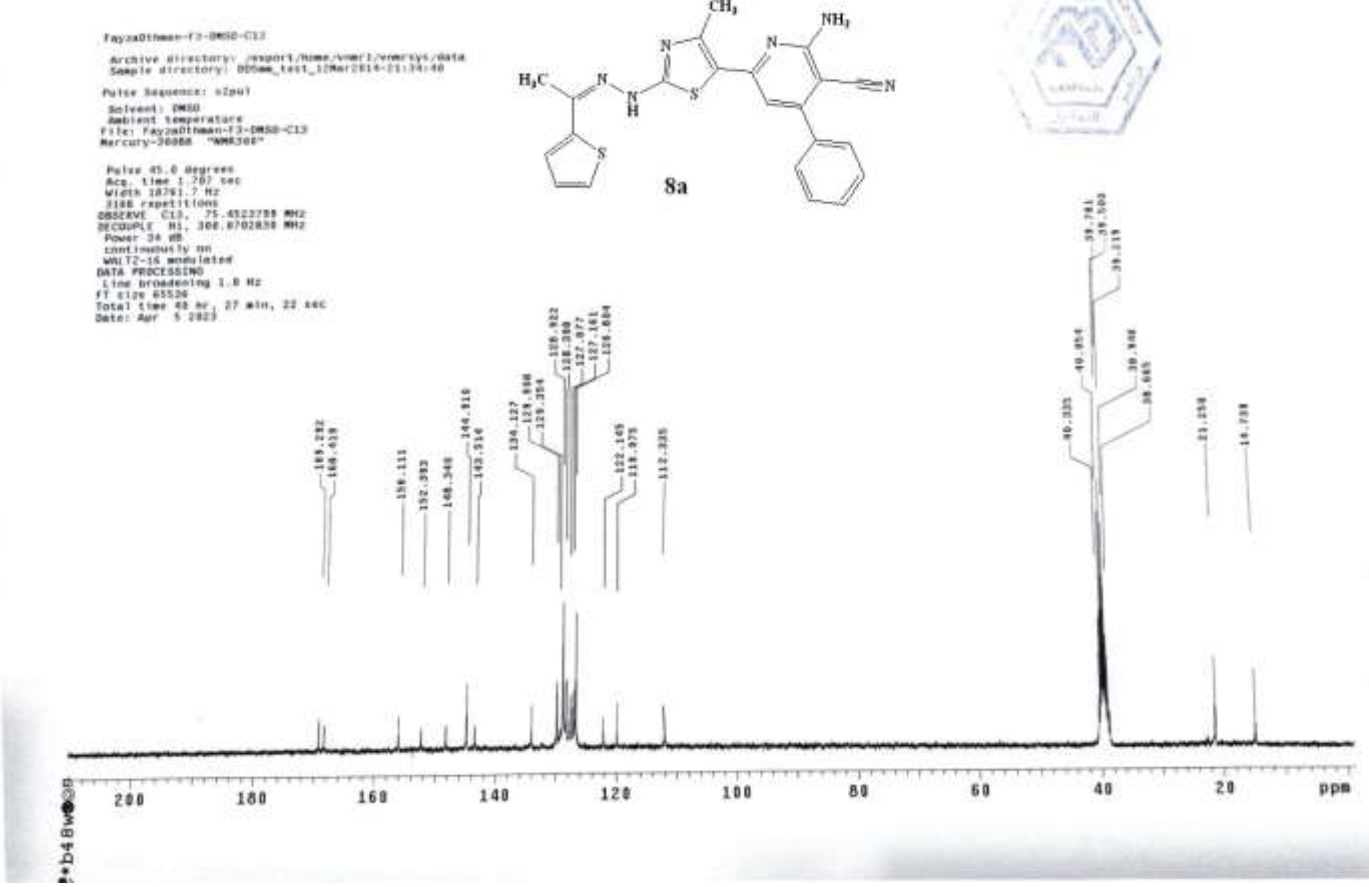

^{13}C-NMR spectra of compound **8a**

Cairo University
Micro Analytical Center

DI Analysis
Shimadzu Qp-2010 Plus

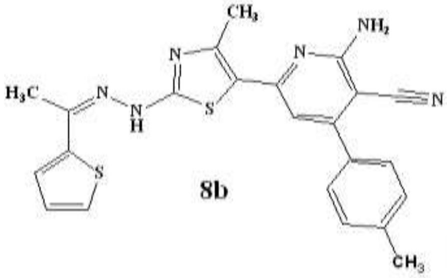

8b

Sample Information

Analyzed by	: Dr. Mai Younis
Analyzed	: 27/08/2019 02:54:27 م
Sample Name	: F4
Sample ID	:
Customer Name	: Dr. Fayza Othman - Science - Cairo
Data File	: C:\GCMSsolution\Data\Project1\F4.QGD
Org Data File	: C:\GCMSsolution\Data\Project1\F4.QGD
Method File	: C:\GCMSsolution\Data\Project1\High Temperature Op
Org Method File	: C:\GCMSsolution\Data\Project1\High Temperature Op
Report File	:
Tuning File	: C:\GCMSsolution\System\Tune1_default.qgt
$EndIf$Modified by	: Dr. Mai Younis
Modified	: 27/08/2019 02:58:40 م

Method

----- Analytical Line 1 -----

IonSourceTemp	:250.00 °C
[MS Table]	
--Group 1 - Event 1--	
Start Time	:0.00min
End Time	:10.00min
ACQ Mode	:Scan
Event Time	:0.50sec
Scan Speed	:1111
Start m/z	:50.00
End m/z	:550.00
Electron Voltage	: 70 eV
Ionization Mode	: EI

C:\GCMSsolution\Data\Project1\F4.QGD

Chromatogram F4 C:\GCMSsolution\Data\Project1\F4.QGD

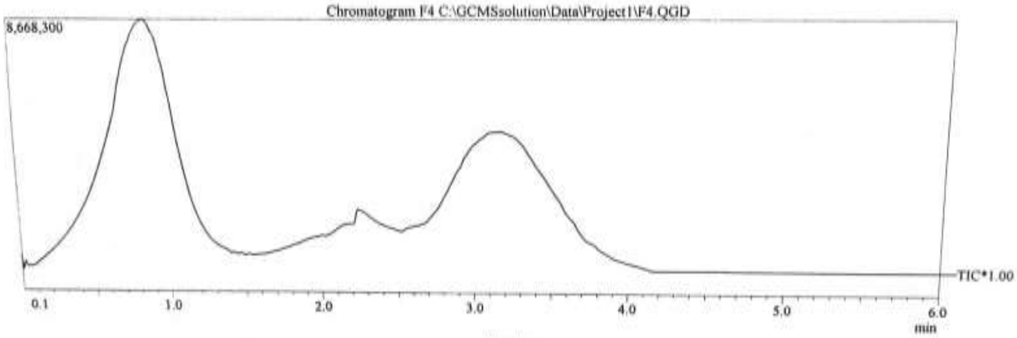

Spectrum

Line#:1 R.Time:3.1(Scan#:375)
MassPeaks:399
RawMode:Single 3.1(375) BasePeak:57(301854)
BG Mode:None Group 1 - Event 1

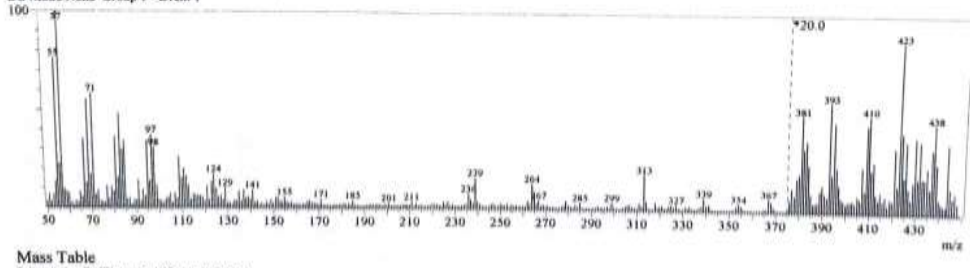

Mass Table
Line#:1 R.Time:3.1(Scan#:375)
MassPeaks:399
RawMode:Single 3.1(375) BasePeak:57(301854)
BG Mode:None Group 1 - Event 1

#	m/z	Abs. In	Rel. Int.	#	m/z	Abs. In	Rel. Int.	#	m/z	Abs. In	Rel. Int.
1	50.00	8705	2.88	4	53.00	19652	6.51	7	56.05	67099	22.23
2	51.00	20052	6.64	5	54.05	40410	13.39	8	57.00	301854	100.00
3	52.00	8433	2.79	6	55.00	229879	76.16	9	58.00	28166	9.33

Mass spectra of compound **8b**

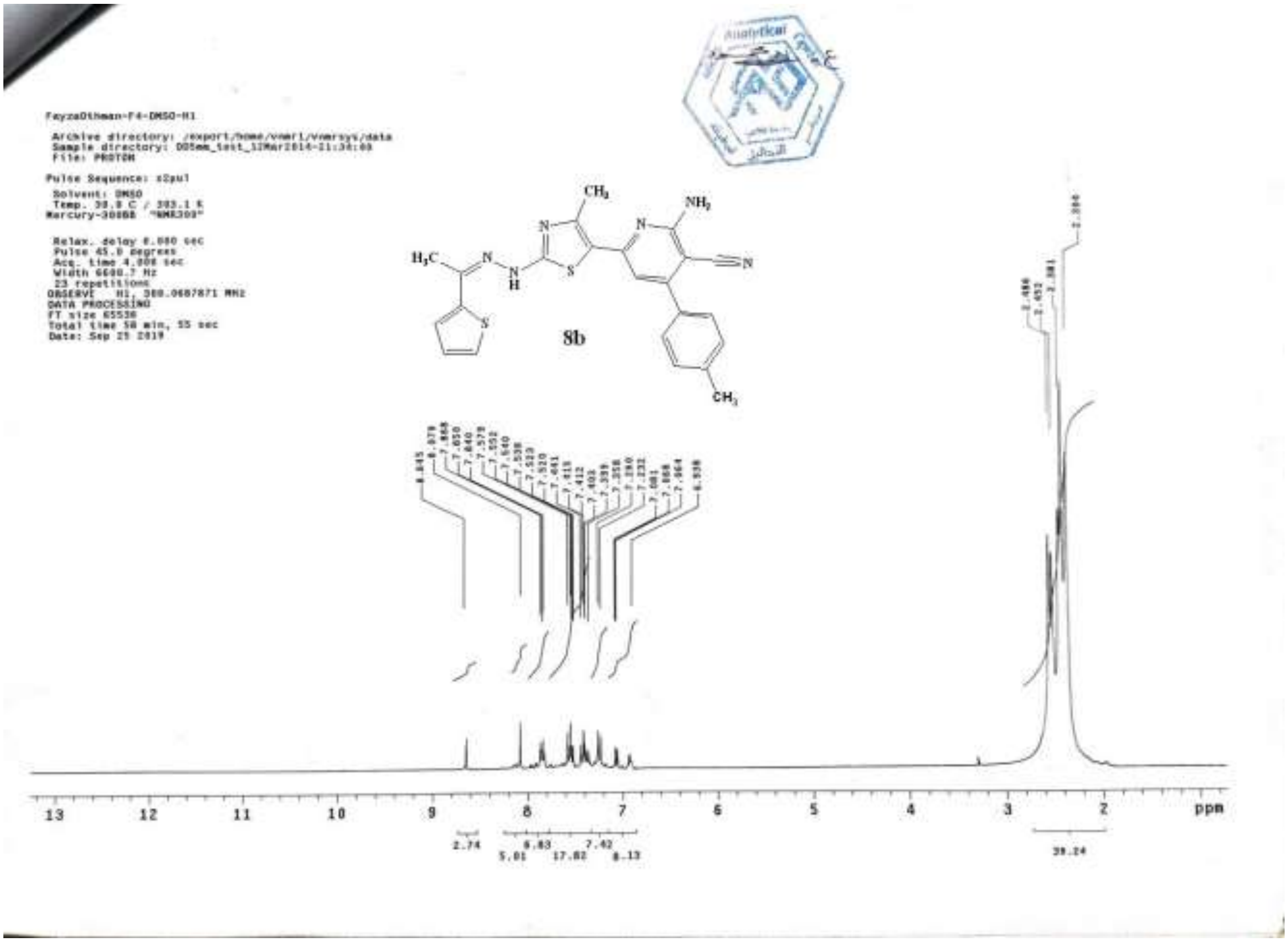

^{1}H-NMR spectra of compound **8b**

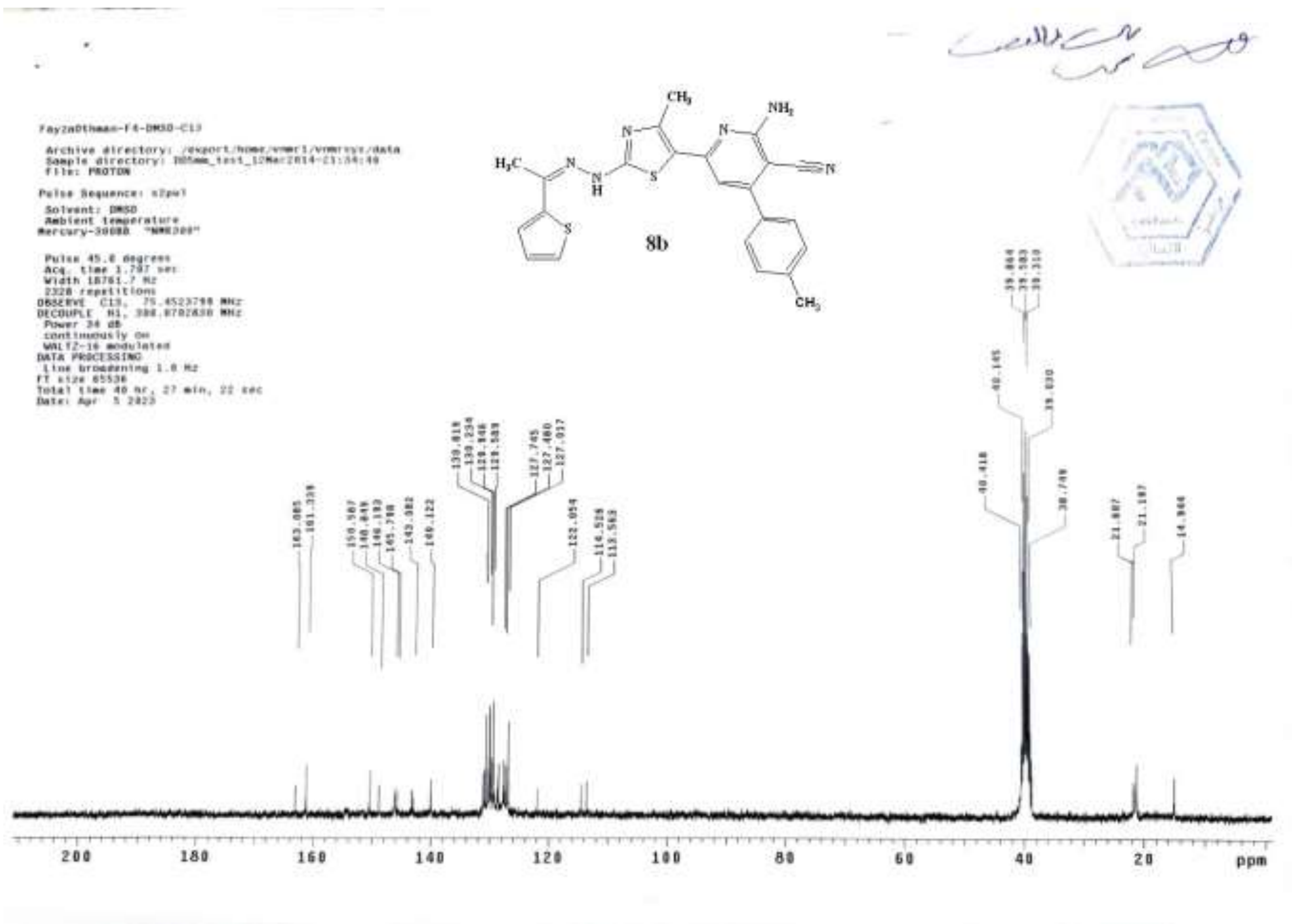

^{13}C-NMR spectra of compound **8b**

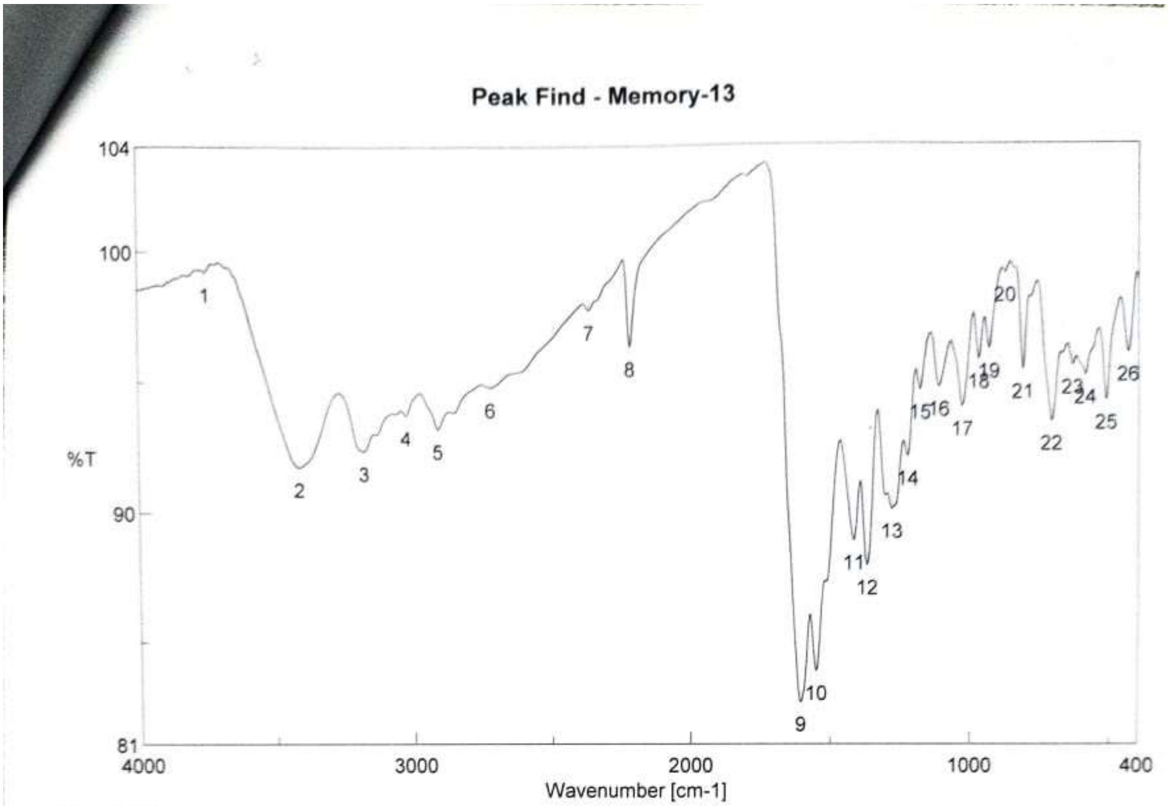

[Comments]

Sample name	F4
Comment	12/2018
User	IR
Division	IR
Company	MAC

[Result of Peak Picking]

No.	Position	Intensity	No.	Position	Intensity	No.	Position	Intensity
1	3754.73	99.2222	2	3419.17	91.7877	3	3186.79	92.4049
4	3034.44	93.7705	5	2918.73	93.2482	6	2727.82	94.8251
7	2370.09	97.7512	8	2221.59	96.3559	9	1605.45	82.6371
10	1550.49	83.849	11	1416.46	88.8668	12	1369.21	87.9095
13	1281.47	90.0695	14	1225.54	92.0897	15	1183.11	94.6143
16	1115.62	94.7216	17	1031.73	93.9622	18	973.876	95.7597
19	937.235	96.1588	20	879.381	99.0589	21	814.777	95.3733
22	710.64	93.4037	23	636.394	95.5344	24	591.075	95.1588
25	515.865	94.2022	26	437.762	96.0332			

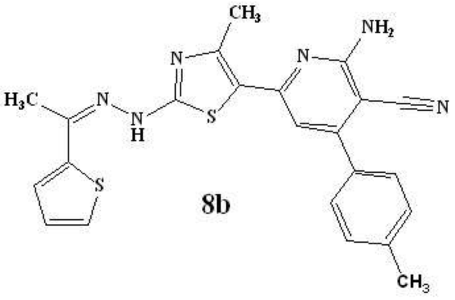

8b

IR spectra of compound **8b**

27-Aug-19 16:13:36

Cairo University
Micro Analytical Center

DI Analysis
Shimadzu Qp-2010 Plus

Sample Information

Analyzed by	: Dr. Mai Younis
Analyzed	: 27/08/2019 04:07:46 م
Sample Name	: F8
Sample ID	:
Customer Name	: Dr. Fayza Othman - Science - Cairo
Data File	: C:\GCMSsolution\Data\Project1\F8.QGD
Org Data File	: C:\GCMSsolution\Data\Project1\F8.QGD
Method File	: C:\GCMSsolution\Data\Project1\High Temperature Op
Org Method File	: C:\GCMSsolution\Data\Project1\High Temperature Op
Report File	:
Tuning File	: C:\GCMSsolution\System\Tune1_default.qgt
$EndIf$Modified by	: Dr. Mai Younis
Modified	: 27/08/2019 04:12:20 م

Method

===== Analytical Line 1 =====

IonSourceTemp	:250.00 °C
[MS Table]	
--Group 1 - Event 1--	
Start Time	:0.00min
End Time	:10.00min
ACQ Mode	:Scan
Event Time	:0.50sec
Scan Speed	:1111
Start m/z	:50.00
End m/z	:550.00
Electron Voltage	: 70 eV
Ionization Mode	: EI

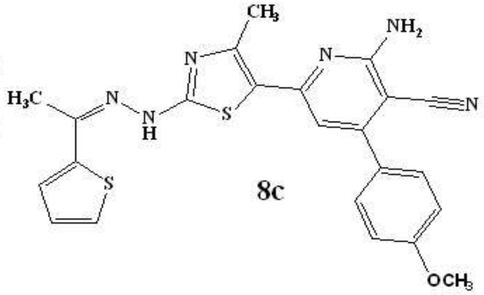

8c

C:\GCMSsolution\Data\Project1\F8.QGD

Chromatogram F8 C:\GCMSsolution\Data\Project1\F8.QGD

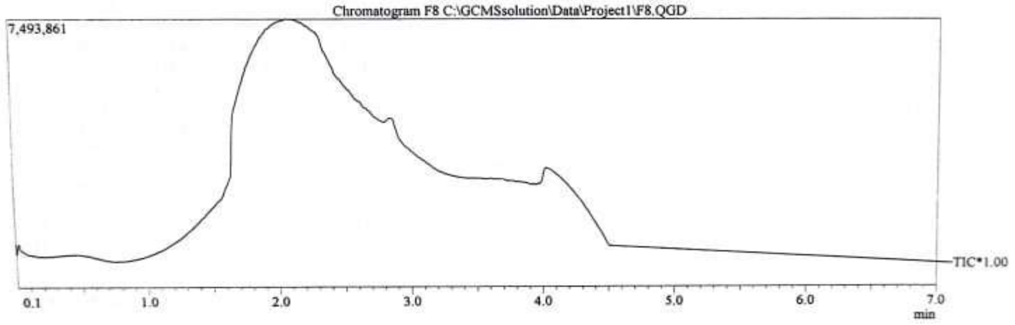

Spectrum

Line#:1 R.Time:3.6(Scan#:435)
MassPeaks:419
RawMode:Single 3.6(435) BasePeak:121(119064)
BG Mode:None Group 1 - Event 1

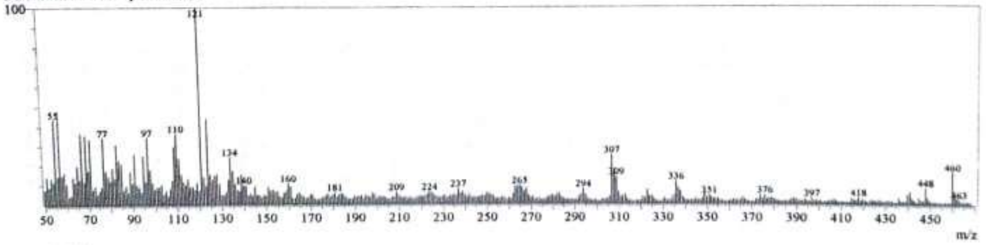

Mass Table
Line#:1 R.Time:3.6(Scan#:435)
MassPeaks:419
RawMode:Single 3.6(435) BasePeak:121(119064)
BG Mode:None Group 1 - Event 1

#	m/z	Abs. In	Rel. Int.	#	m/z	Abs. In	Rel. Int.	#	m/z	Abs. In	Rel. Int.
1	50.00	8631	7.25	4	53.00	15959	13.40	7	56.05	16792	14.10
2	51.00	16697	14.02	5	54.05	13674	11.48	8	57.00	56788	47.70
3	52.00	10374	8.71	6	55.00	51638	43.37	9	58.00	17378	14.60

Mass spectra of compound **8c**

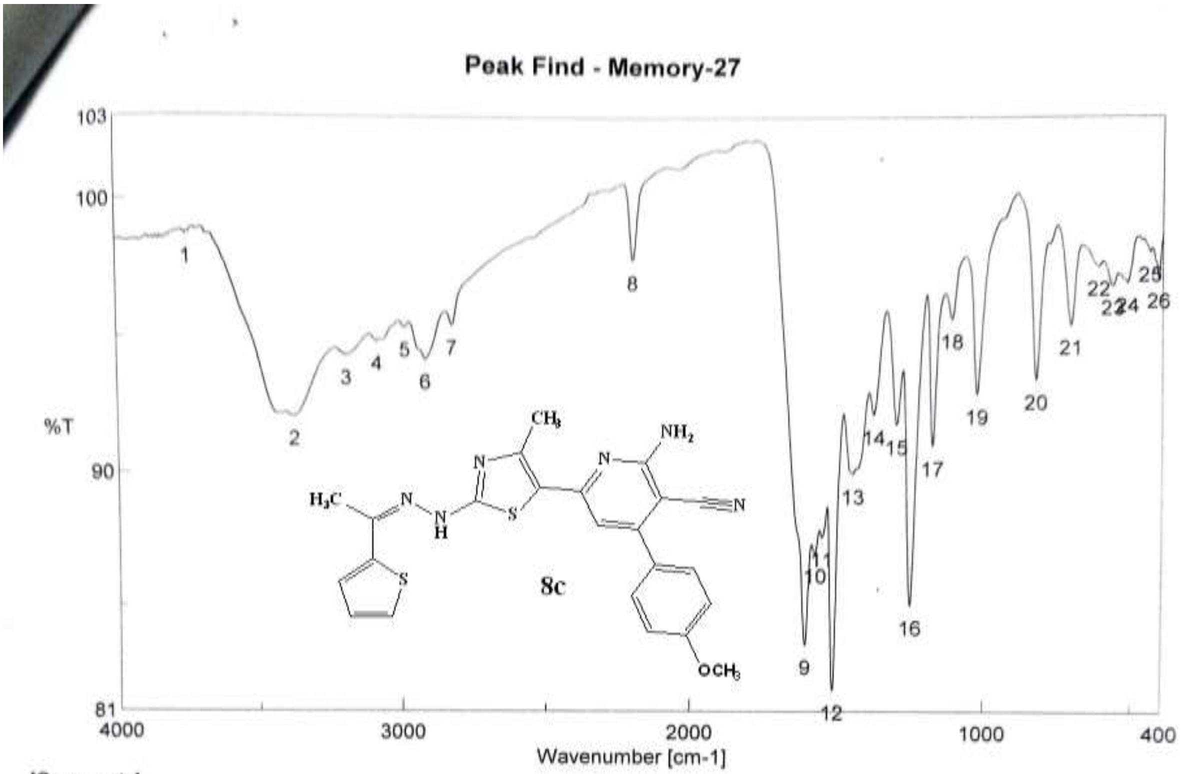

[Comments]
Sample name F8
Comment 12/2018
User IR
Division IR
Company MAC

[Result of Peak Picking]

No.	Position	Intensity	No.	Position	Intensity	No.	Position	Intensity
1	3752.8	98.7361	2	3383.5	92.0907	3	3204.15	94.3546
4	3097.12	94.8811	5	3000.69	95.3727	6	2928.38	94.1936
7	2837.74	95.4513	8	2208.09	97.7665	9	1605.45	83.4802
10	1572.66	86.8829	11	1549.52	87.4896	12	1511.92	81.7909
13	1446.35	89.8177	14	1376.93	91.9994	15	1299.79	91.6309
16	1249.65	84.9454	17	1177.33	90.8359	18	1115.62	95.5094
19	1028.84	92.7327	20	828.277	93.2543	21	710.64	95.2878
22	617.109	97.4659	23	569.862	96.7562	24	519.722	96.8541
25	442.583	97.9948	26	413.656	97.0177			

IR spectra of compound **8c**

27-Aug-19 15:56:29

[illegible]

Cairo University
Micro Analytical Center

DI Analysis
Shimadzu Qp-2010 Plus

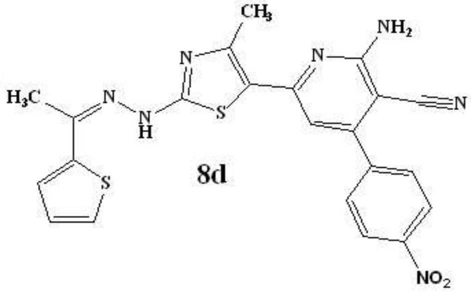

Sample Information

Analyzed by	: Dr. Mai Younis
Analyzed	: 27/08/2019 03:49:36 م
Sample Name	: F7
Sample ID	:
Customer Name	: Dr. Fayza Othman - Science - Cairo
Data File	: C:\GCMSsolution\Data\Project1\F7.QGD
Org Data File	: C:\GCMSsolution\Data\Project1\F7.QGD
Method File	: C:\GCMSsolution\Data\Project1\High Temperature Op
Org Method File	: C:\GCMSsolution\Data\Project1\High Temperature Op
Report File	:
Tuning File	: C:\GCMSsolution\System\Tune1_default.qgt
$EndIf$Modified by	: Dr. Mai Younis
Modified	: 27/08/2019 03:54:59 م

Method

===== Analytical Line 1 =====

IonSourceTemp	:250.00 °C
[MS Table]	
--Group 1 - Event 1--	
Start Time	:0.00min
End Time	:10.00min
ACQ Mode	:Scan
Event Time	:0.50sec
Scan Speed	:1111
Start m/z	:50.00
End m/z	:550.00
Electron Voltage	: 70 eV
Ionization Mode	: EI

C:\GCMSsolution\Data\Project1\F7.QGD

Chromatogram F7 C:\GCMSsolution\Data\Project1\F7.QGD

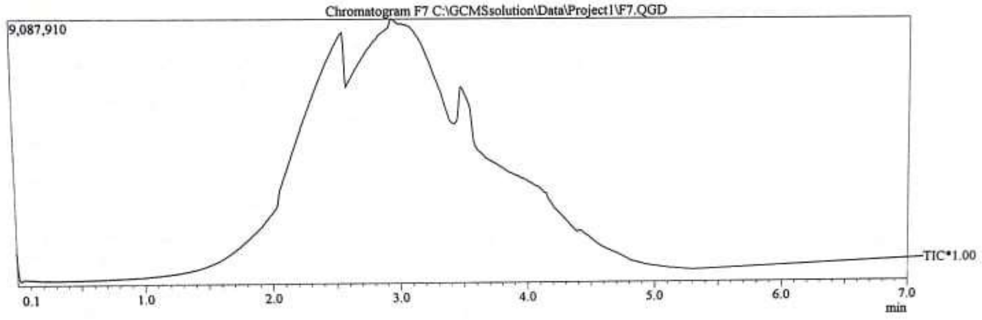

Spectrum

Line#:1 R.Time:4.5(Scan#:540)
MassPeaks:382
RawMode:Single 4.5(540) BasePeak:57(79712)
BG Mode:None Group 1 - Event 1

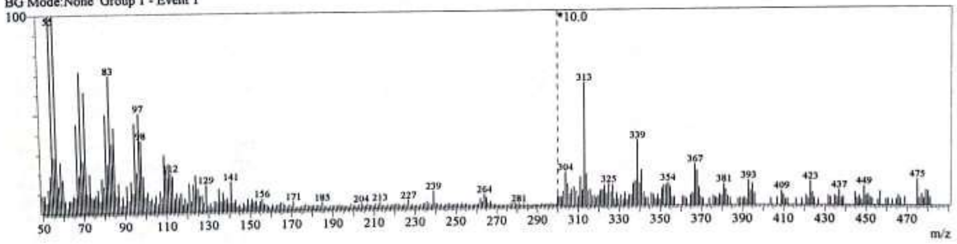

Mass Table
Line#:1 R.Time:4.5(Scan#:540)
MassPeaks:382
RawMode:Single 4.5(540) BasePeak:57(79712)
BG Mode:None Group 1 - Event 1

#	m/z	Abs. Int.	Rel. Int.	#	m/z	Abs. Int.	Rel. Int.	#	m/z	Abs. Int.	Rel. Int.
1	50.00	7033	8.82	4	53.00	9834	12.34	7	56.05	22635	28.40
2	51.00	7410	9.30	5	54.05	15173	19.03	8	57.05	79712	100.00
3	52.00	4198	5.27	6	55.00	79270	99.45	9	58.00	10714	13.44

Mass spectra of compound **8d**

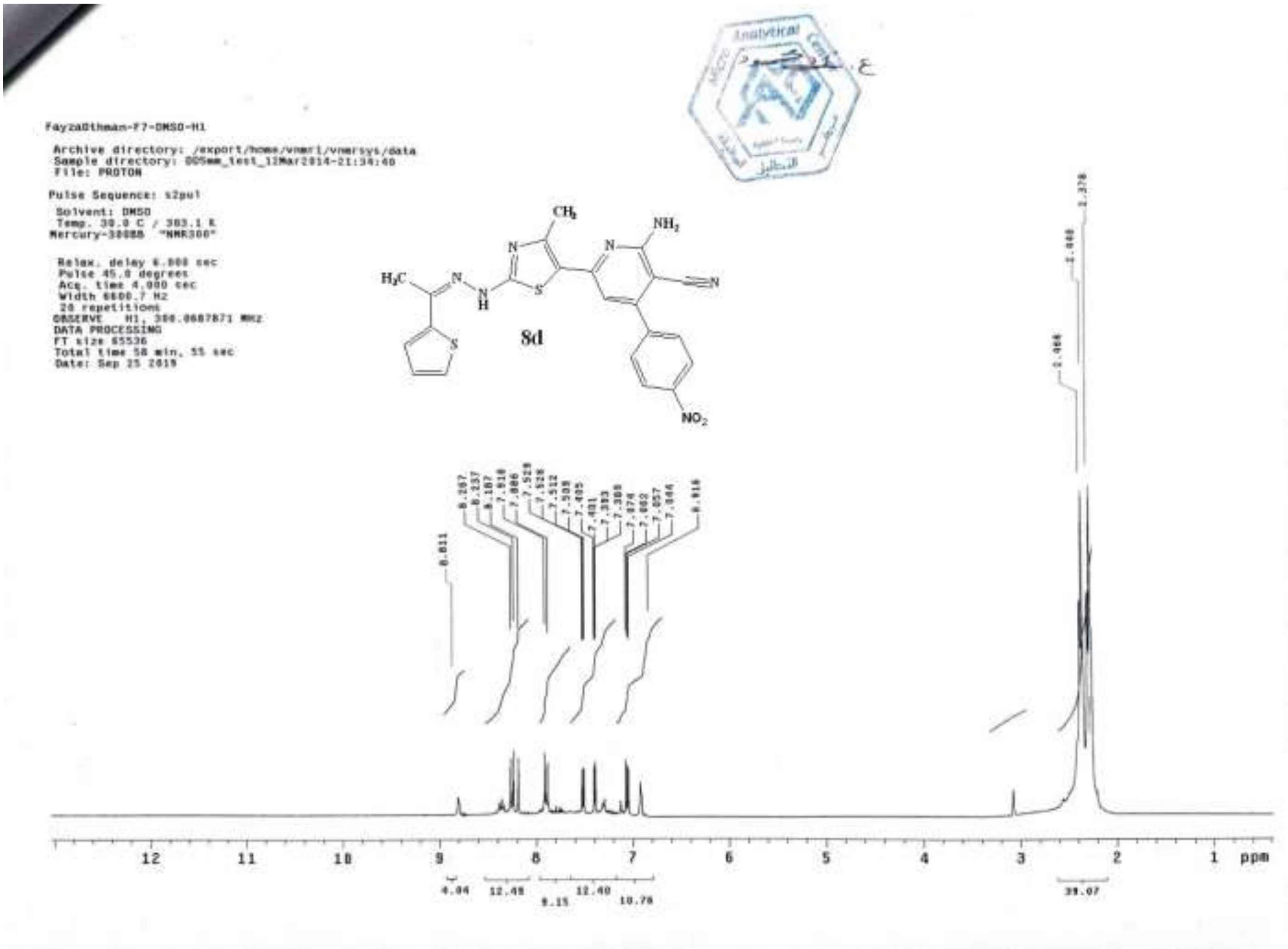

^{1}H-NMR spectra of compound **8d**

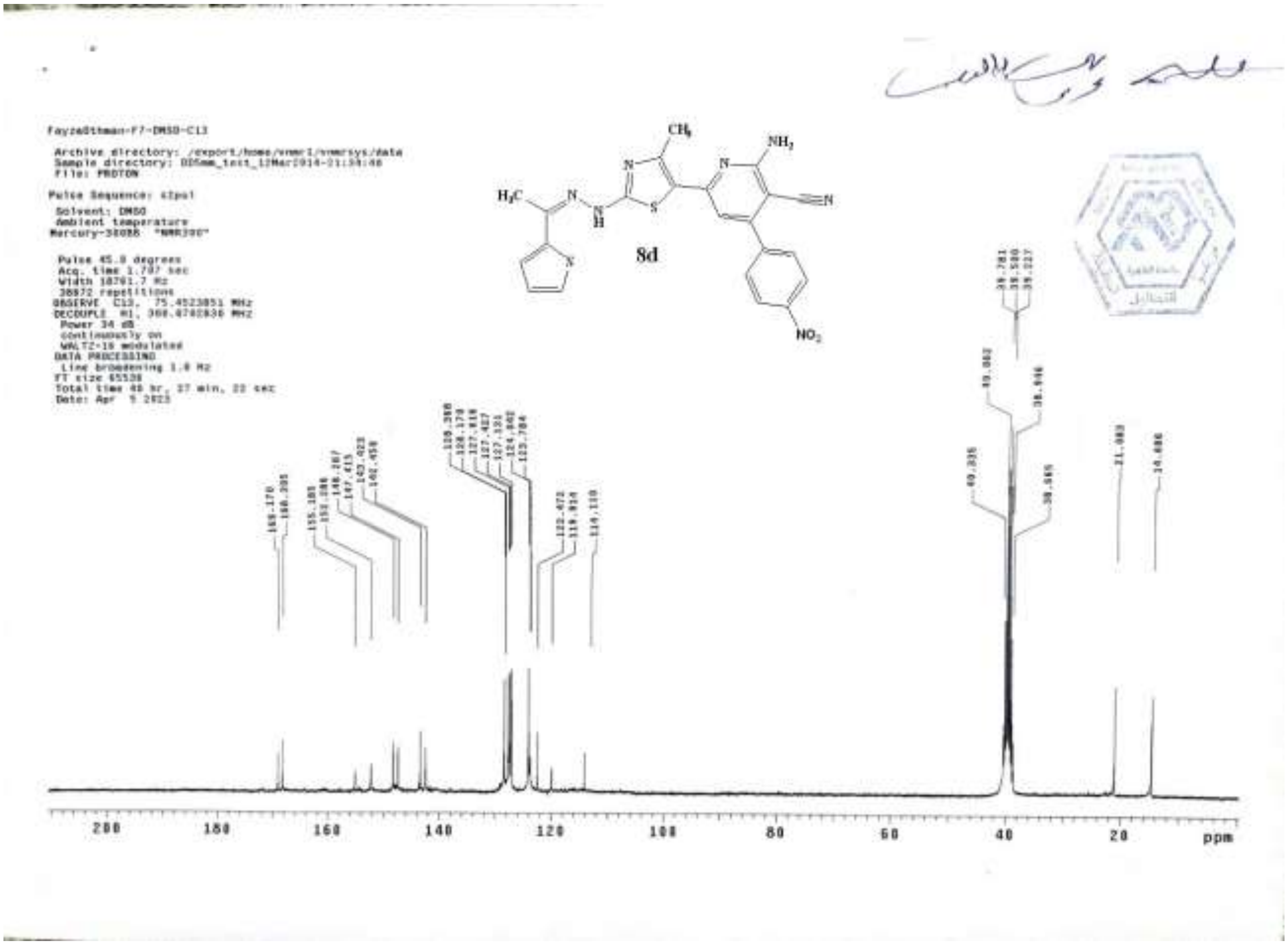

^{13}C-NMR spectra of compound **8d**

Peak Find - Memory-24

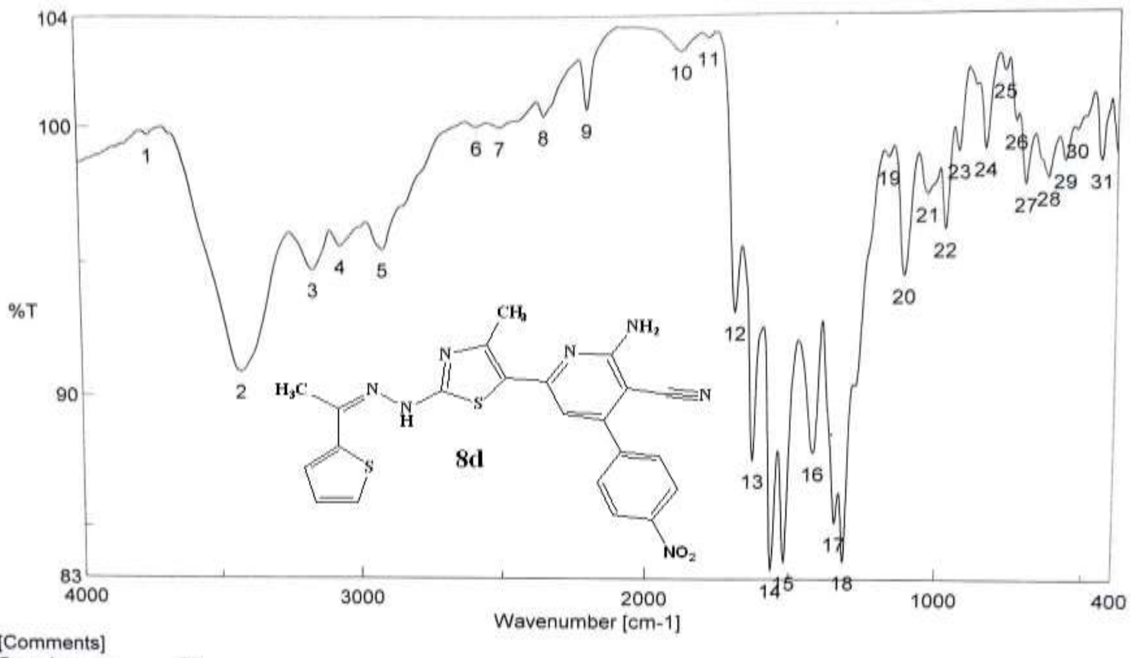

[Comments]
Sample name F7
Comment 12/2018
User IR
Division IR
Company MAC

[Result of Peak Picking]

No.	Position	Intensity	No.	Position	Intensity	No.	Position	Intensity
1	3751.83	99.7013	2	3430.74	90.9116	3	3177.15	94.7356
4	3079.76	95.6042	5	2931.27	95.4752	6	2597.64	99.9606
7	2517.61	99.9206	8	2363.34	100.315	9	2211.95	100.557
10	1887	102.662	11	1793.47	103.134	12	1692.23	93.0308
13	1628.59	87.4969	14	1563.02	83.3527	15	1518.67	83.6492
16	1422.24	87.7705	17	1346.07	85.1311	18	1316.18	83.6762
19	1176.36	98.682	20	1117.55	94.3597	21	1042.34	97.3302
22	980.625	96.0419	23	937.235	98.894	24	846.597	98.9821
25	782.958	101.879	26	743.424	99.9869	27	709.676	97.6644
28	632.537	97.8978	29	576.612	98.5293	30	538.042	99.6529
31	453.19	98.5312						

IR spectra of compound **8d**

27-Aug-19 15:29:57

Cairo University
Micro Analytical Center

DI Analysis
Shimadzu Qp-2010 Plus

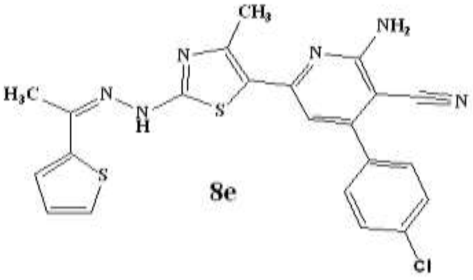

Sample Information

Analyzed by	: Dr. Mai Younis
Analyzed	: 27/08/2019 03:23:43 م
Sample Name	: F5
Sample ID	:
Customer Name	: Dr. Fayza Othman - Science - Cairo
Data File	: C:\GCMSsolution\Data\Project1\F5..QGD
Org Data File	: C:\GCMSsolution\Data\Project1\F5..QGD
Method File	: C:\GCMSsolution\Data\Project1\High Temperature Op
Org Method File	: C:\GCMSsolution\Data\Project1\High Temperature Op
Report File	:
Tuning File	: C:\GCMSsolution\System\Tune1_default.qgt
$EndIf$Modified by	: Dr. Mai Younis
Modified	: 27/08/2019 03:28:15 م

Method

===== Analytical Line 1 =====

IonSourceTemp	:250.00 °C
[MS Table]	
--Group 1 - Event 1--	
Start Time	:0.00min
End Time	:10.00min
ACQ Mode	:Scan
Event Time	:0.50sec
Scan Speed	:1111
Start m/z	:50.00
End m/z	:550.00
Electron Voltage	: 70 eV
Ionization Mode	: EI

C:\GCMSsolution\Data\Project1\F5..QGD

Chromatogram F5. C:\GCMSsolution\Data\Project1\F5..QGD

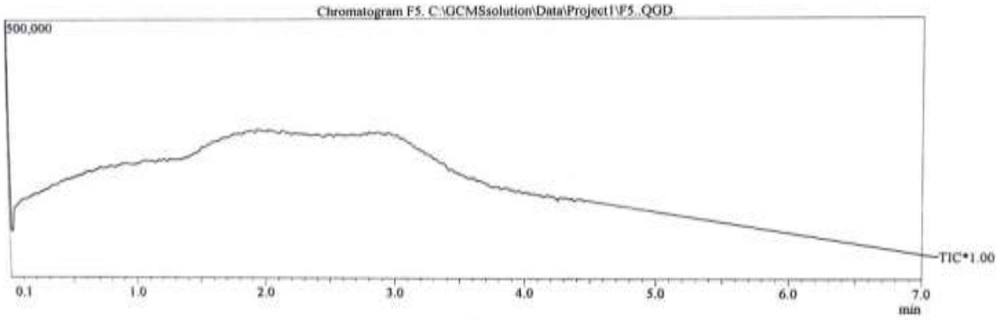

Spectrum

Line#:1 R.Time:3.0(Scan#:360)
MassPeaks:238
RawMode:Single 3.0(360) BasePeak:141(8586)
BG Mode:None Group 1 - Event 1

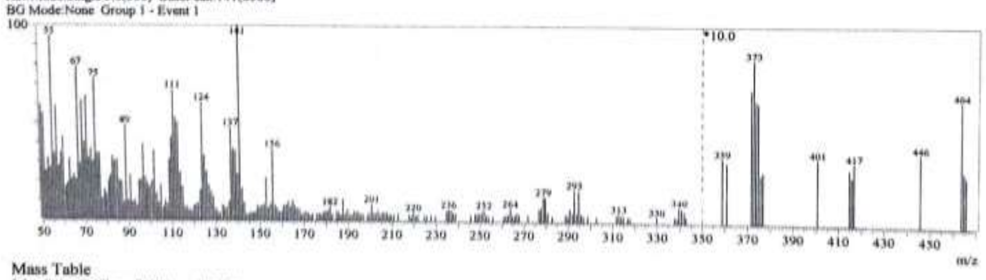

Mass Table
Line#:1 R.Time:3.0(Scan#:360)
MassPeaks:238
RawMode:Single 3.0(360) BasePeak:141(8586)
BG Mode:None Group 1 - Event 1

#	m/z	Abs. In	Rel. Int.	#	m/z	Abs. In	Rel. Int.	#	m/z	Abs. In	Rel. Int.
1	50.00	5084	59.21	4	53.00	2718	31.66	7	56.00	2951	34.37
2	51.00	4746	55.28	5	54.00	2297	26.75	8	57.00	5052	58.84
3	52.00	2145	24.98	6	55.00	8103	94.37	9	58.00	2366	27.56

Mass spectra of compound **8e**

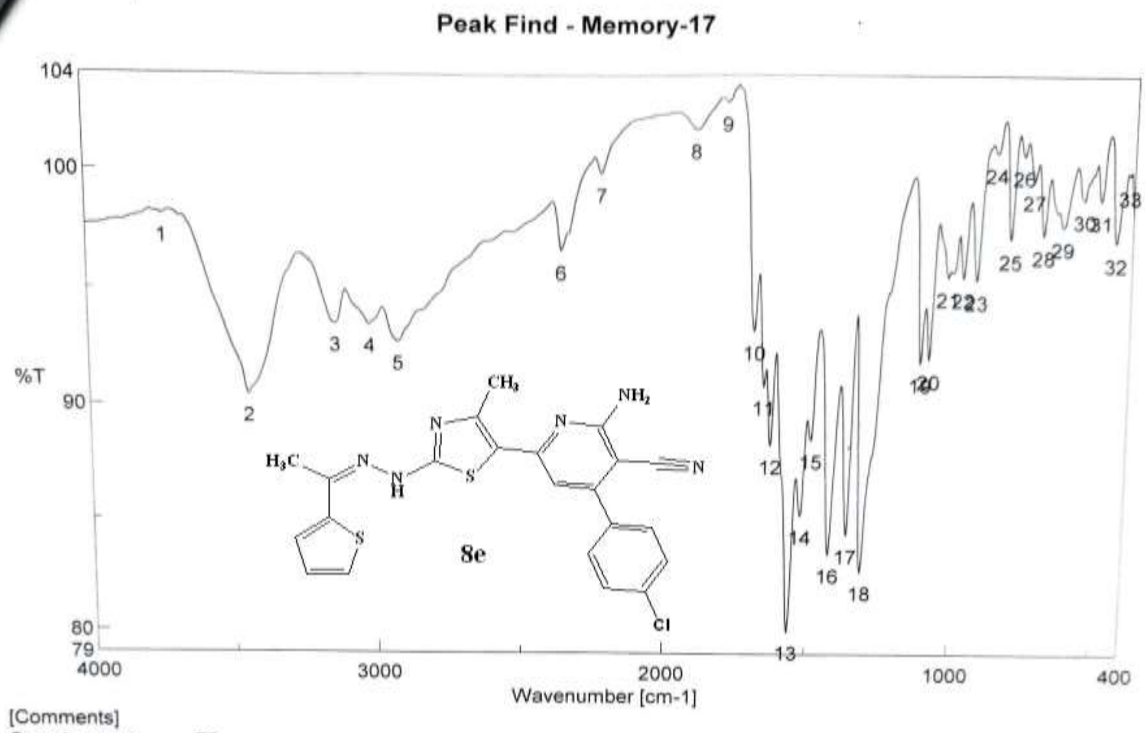

[Comments]

Sample name	F5
Comment	12/2018
User	IR
Division	IR
Company	MAC

[Result of Peak Picking]

No.	Position	Intensity	No.	Position	Intensity	No.	Position	Intensity
1	3737.37	98.111	2	3448.1	90.4613	3	3148.22	93.5255
4	3028.66	93.4981	5	2930.31	92.7467	6	2361.41	96.6073
7	2222.56	99.8616	8	1899.54	101.704	9	1793.47	102.864
10	1691.27	93.1892	11	1655.59	90.8177	12	1630.52	88.2357
13	1563.99	79.9987	14	1523.49	85.1621	15	1490.7	88.4746
16	1425.14	83.4537	17	1363.43	84.3269	18	1312.32	82.6794
19	1120.44	91.8121	20	1091.51	91.9535	21	1032.69	95.4492
22	980.625	95.4261	23	934.342	95.3219	24	874.56	100.713
25	821.527	97.0473	26	780.065	100.615	27	744.388	99.5848
28	707.747	97.2201	29	642.179	97.5914	30	569.862	98.7582
31	512.972	98.7272	32	458.011	96.9321	33	416.549	99.8152

IR spectra of compound **8e**

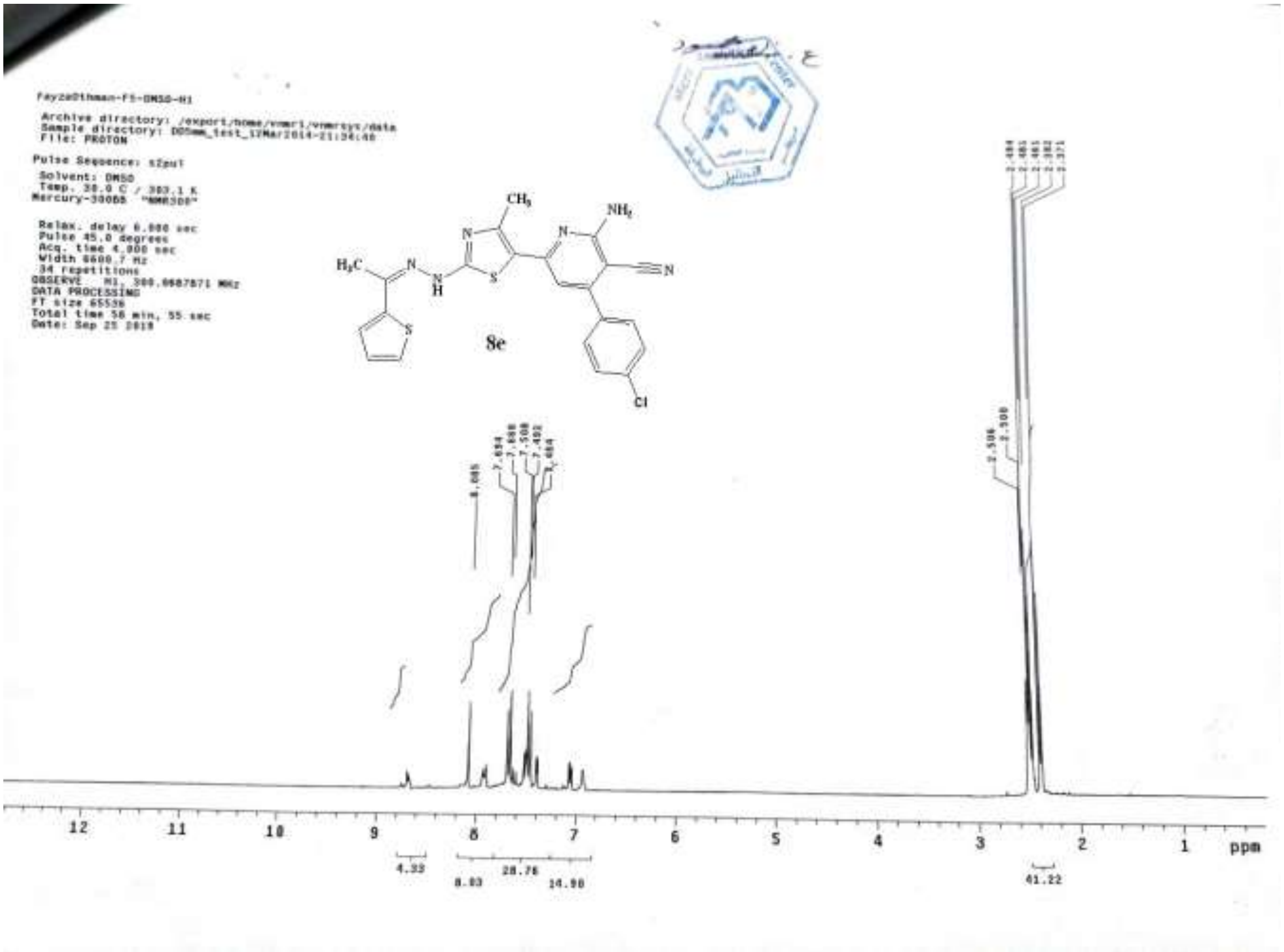

^{1}H-NMRspectra of compound **8e**

27-Aug-19 15:48:14

ع. شريح سود

Cairo University
Micro Analytical Center

DI Analysis
Shimadzu Qp-2010 Plus

Sample Information

Analyzed by	: Dr. Mai Younis
Analyzed	: 27/08/2019 03:41:03 م
Sample Name	: F6
Sample ID	:
Customer Name	: Dr. Fayza Othman - Science - Cairo
Data File	: C:\GCMSsolution\Data\Project1\F6.QGD
Org Data File	: C:\GCMSsolution\Data\Project1\F6.QGD
Method File	: C:\GCMSsolution\Data\Project1\High Temperature Op
Org Method File	: C:\GCMSsolution\Data\Project1\High Temperature Op
Report File	:
Tuning File	: C:\GCMSsolution\System\Tune1_default.qgt
$EndIf$Modified by	: Dr. Mai Younis
Modified	: 27/08/2019 03:45:30 م

Method

====== Analytical Line 1 ======

IonSourceTemp	:250.00 °C
[MS Table]	
--Group 1 - Event 1--	
Start Time	:0.00min
End Time	:10.00min
ACQ Mode	:Scan
Event Time	:0.50sec
Scan Speed	:1111
Start m/z	:50.00
End m/z	:550.00
Electron Voltage	: 70 eV
Ionization Mode	: EI

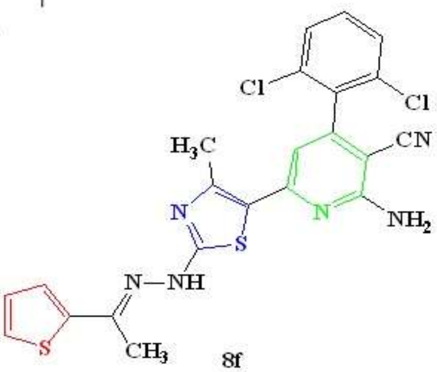

8f

C:\GCMSsolution\Data\Project1\F6.QGD

Chromatogram F6 C:\GCMSsolution\Data\Project1\F6.QGD

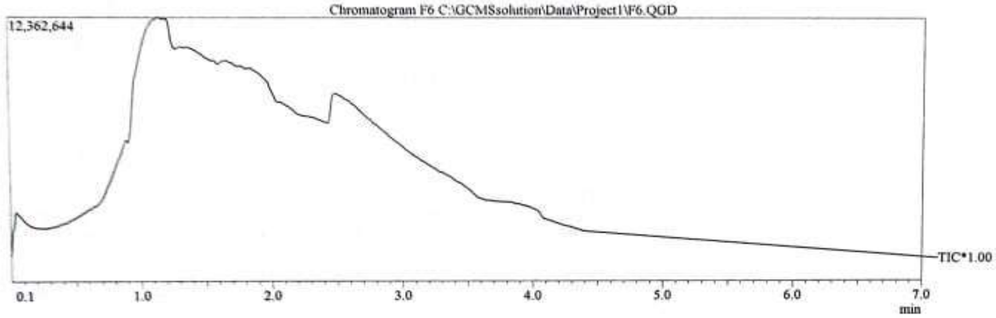

Spectrum

Line#:1 R.Time:2.8(Scan#:334)
MassPeaks:455
RawMode:Single 2.8(334) BasePeak:308(311107)
BG Mode:None Group 1 - Event 1

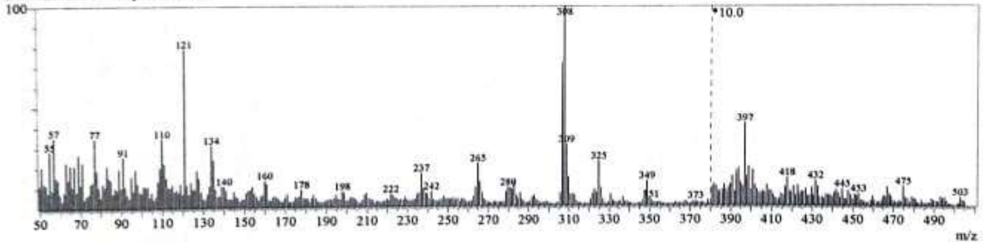

Mass Table
Line#:1 R.Time:2.8(Scan#:334)
MassPeaks:455
RawMode:Single 2.8(334) BasePeak:308(311107)
BG Mode:None Group 1 - Event 1

#	m/z	Abs. Int	Rel. Int.	#	m/z	Abs. Int	Rel. Int.	#	m/z	Abs. Int	Rel. Int.
1	50.00	34670	11.14	4	53.00	35970	11.56	7	56.05	27516	8.84
2	51.00	63222	20.32	5	54.05	23464	7.54	8	57.00	108273	34.80
3	52.00	37692	12.12	6	55.00	87274	28.05	9	57.95	46554	14.96

Mass spectra of compound **8f**

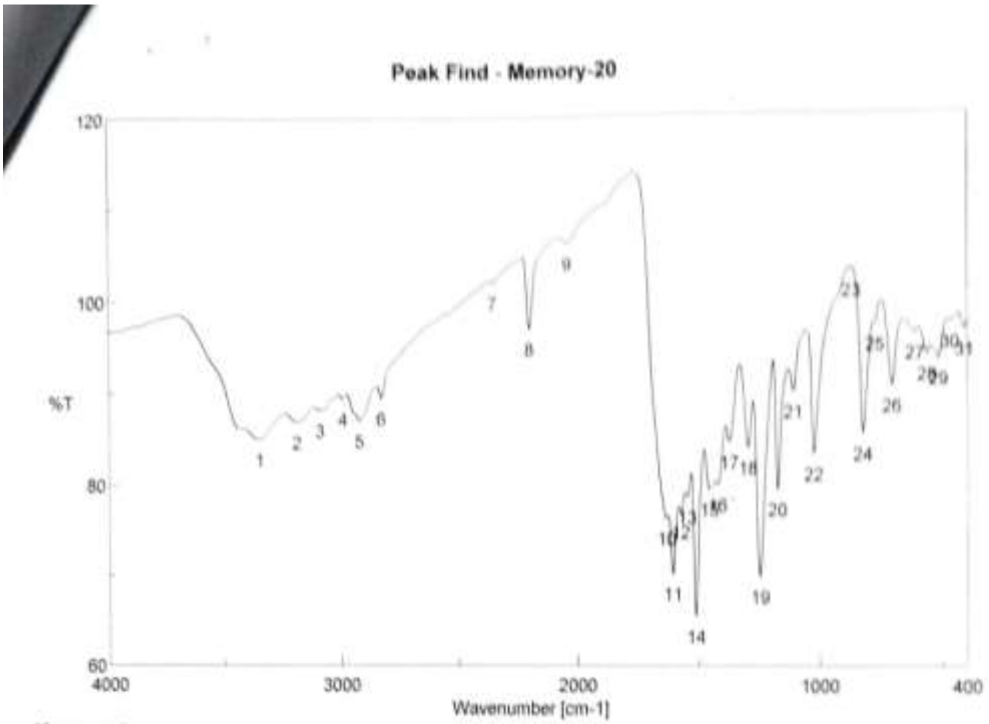

[Comments]

Sample name	F6
Comment	12/2018
User	IR
Division	IR
Company	MAC

[Result of Peak Picking]

No.	Position	Intensity	No.	Position	Intensity	No.	Position	Intensity
1	3353.6	85.0103	2	3194.51	86.8864	3	3096.15	88.1441
4	2999.73	89.4215	5	2928.38	87.007	6	2835.81	89.461
7	2364.3	101.907	8	2207.13	96.8547	9	2049.96	106.068
10	1632.45	76.0639	11	1605.45	70.0397	12	1573.63	76.7853
13	1548.56	78.3886	14	1510.95	65.4291	15	1455.99	79.2056
16	1421.28	79.6748	17	1375	84.2947	18	1297.86	83.6832
19	1247.72	69.6847	20	1176.36	79.1675	21	1113.69	89.7068
22	1027.87	82.9717	23	877.452	102.78	24	827.312	85.0971
25	780.065	96.9508	26	709.676	90.2889	27	615.181	96.0077
28	566.005	93.7328	29	517.793	93.2679	30	471.51	97.272
31	411.728	96.4427						

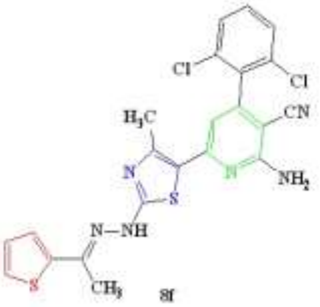

8f

IR spectra of compound **8f**
